# LGBTQ + cultural competency training for health professionals: a systematic review

**DOI:** 10.1186/s12909-023-04373-3

**Published:** 2023-08-09

**Authors:** Hyunmin Yu, Dalmacio Dennis Flores, Stephen Bonett, José Arturo Bauermeister

**Affiliations:** School of Nursing, University of Pennsylvania, 418 Curie Blvd, Philadelphia, PA 19104 USA

**Keywords:** LGBTQ + health, Cultural competence, Cultural competency training, Health professionals, Healthcare providers

## Abstract

**Background:**

Health disparities experienced by LGBTQ + individuals have been partially attributed to health professionals’ lack of cultural competence to work with them. Cultural competence, the intricate integration of knowledge, skills, attitudes, and behaviors that improve cross-cultural communication and interpersonal relationships, has been used as a training framework to enhance interactions between LGBTQ + patients and health professionals. Despite multiple published LGBTQ + cultural competency trainings, there has been no quantitative appraisal and synthesis of them. This systematic review assessed articles evaluating the design and effectiveness of these trainings and examined the magnitude of their effect on cultural competence outcomes.

**Methods:**

Included studies quantitatively examined the effectiveness of LGBTQ + cultural competency trainings for health professionals across all disciplines in various healthcare settings. 2,069 citations were retrieved from five electronic databases with 44 articles meeting inclusion criteria. The risk of bias in the included studies was assessed by two authors utilizing the Joanna Briggs Institute critical appraisal checklists. Data extracted included study design, country/region, sample characteristic, training setting, theoretical framework, training topic, modality, duration, trainer, training target, measurement instrument, effect size and key findings. This review followed the PRISMA statement and checklist to ensure proper reporting.

**Results:**

75% of the studies were published between 2017 and 2023. Four study designs were used: randomized controlled trial (*n* = 1), quasi-experimental pretest–posttest without control (*n* = 39), posttest only with control (*n* = 1) and posttest only without control (*n* = 3). Training modalities were multiple modalities with (*n* = 9) and without simulation (*n* = 25); single modality with simulation (*n* = 1); and with didactic lectures (*n* = 9). Trainings averaged 3.2 h. Ten studies employed LGBTQ + trainers. The training sessions resulted in statistically significant improvements in the following cultural competence constructs: (1) knowledge of LGBTQ + culture and health (*n* = 28, effect size range = 0.28 – 1.49), (2) skills to work with LGBTQ + clients (*n* = 8, effect size range = 0.12 – 1.12), (3) attitudes toward LGBTQ + individuals (*n* = 14, effect size range = 0.19 – 1.03), and (4) behaviors toward LGBTQ + affirming practices (*n* = 7, effect size range = 0.51 – 1.11).

**Conclusions:**

The findings of this review highlight the potential of LGBTQ + cultural competency training to enhance cultural competence constructs, including (1) knowledge of LGBTQ + culture and health, (2) skills to work with LGBTQ + clients, (3) attitudes toward LGBTQ + individuals, and (4) behaviors toward LGBTQ + affirming practices, through an interdisciplinary and multi-modal approach. Despite the promising results of LGBTQ + cultural competency training in improving health professionals’ cultural competence, there are limitations in study designs, sample sizes, theoretical framing, and the absence of longitudinal assessments and patient-reported outcomes, which call for more rigorous research. Moreover, the increasing number of state and federal policies that restrict LGBTQ + health services highlight the urgency of equipping health professionals with culturally responsive training. Organizations and health systems must prioritize organizational-level changes that support LGBTQ + inclusive practices to provide access to safe and affirming healthcare services for LGBTQ + individuals.

## Introduction

In 2022, Gallup estimates that 7.1% of American adults, including 20.8% of Generation Z individuals born between 1997 and 2003, self-identify as lesbian, gay, bisexual, transgender, queer or questioning and others (LGBTQ +), often referred to as sexual and gender minorities or sexual and gender diverse groups, and that percentage has doubled since 2012 [1]. Despite improved societal attitudes toward LGBTQ + persons over the last several decades [2, 3], health disparities that adversely affect LGBTQ + people persist [4]. Compared to their heterosexual and cisgender peers, LGBTQ + individuals healthcare avoidance and/or distrust of health professionals, due to previous or anticipated stigmatization and/or discrimination during healthcare encounters, including outright refusals of care [5–7]. These disparities associated with social and structural inequities have a direct impact on LGBTQ + clients’ negative health outcomes, including sexual and reproductive health, mental health, cardiovascular and cancer-related outcomes [4, 8].

A lack of clinically and culturally responsive healthcare providers remains a major concern for LGBTQ + patients according to the National Academies of Sciences, Engineering, and Medicine [4]. To this end, national organizations have developed and issued protocols, such as the Joint Commission’s field guide [9] and the Fenway Guide [10], to assist healthcare institutions and professionals in providing more LGBTQ + affirming care. Despite such initiatives, there is a widespread scarcity of LGBTQ + focused trainings to equip health professionals with clinical and cultural competence to address the frequently unmet and unique health needs of LGBTQ + patients, such as gender-affirming treatments for transgender patients and sexually transmitted infection screening for men who have sex with men [4, 11, 12]. According to the 2015 U.S. Transgender Survey, 24% of transgender patients have had to teach their providers about their health needs [13], causing them to feel frustrated, unsafe, anxious and/or burdened [14].

Likewise, health professionals have admitted their lack of training regarding LGBTQ + care [15]. A recent national survey also indicates that a lack of LGBTQ + specific training was a major barrier for healthcare providers to provide LGBTQ + care [16]. Also, more than 70% of primary care providers in a cross-sectional study who practice in Indiana described inadequate training on health needs and clinical management for LGBTQ + clients [17]. From two U.S.-based nationwide surveys, 80.6% of endocrinologists and 82.5% emergency physicians expressed never receiving training for transgender care although 80% and 88% respectively have treated a transgender patient [18, 19]. Similarly, 79% of nurses in a study who practice in San Francisco reported that they have not received LGBTQ + training from their organizations [20].

**Fig. 1:**
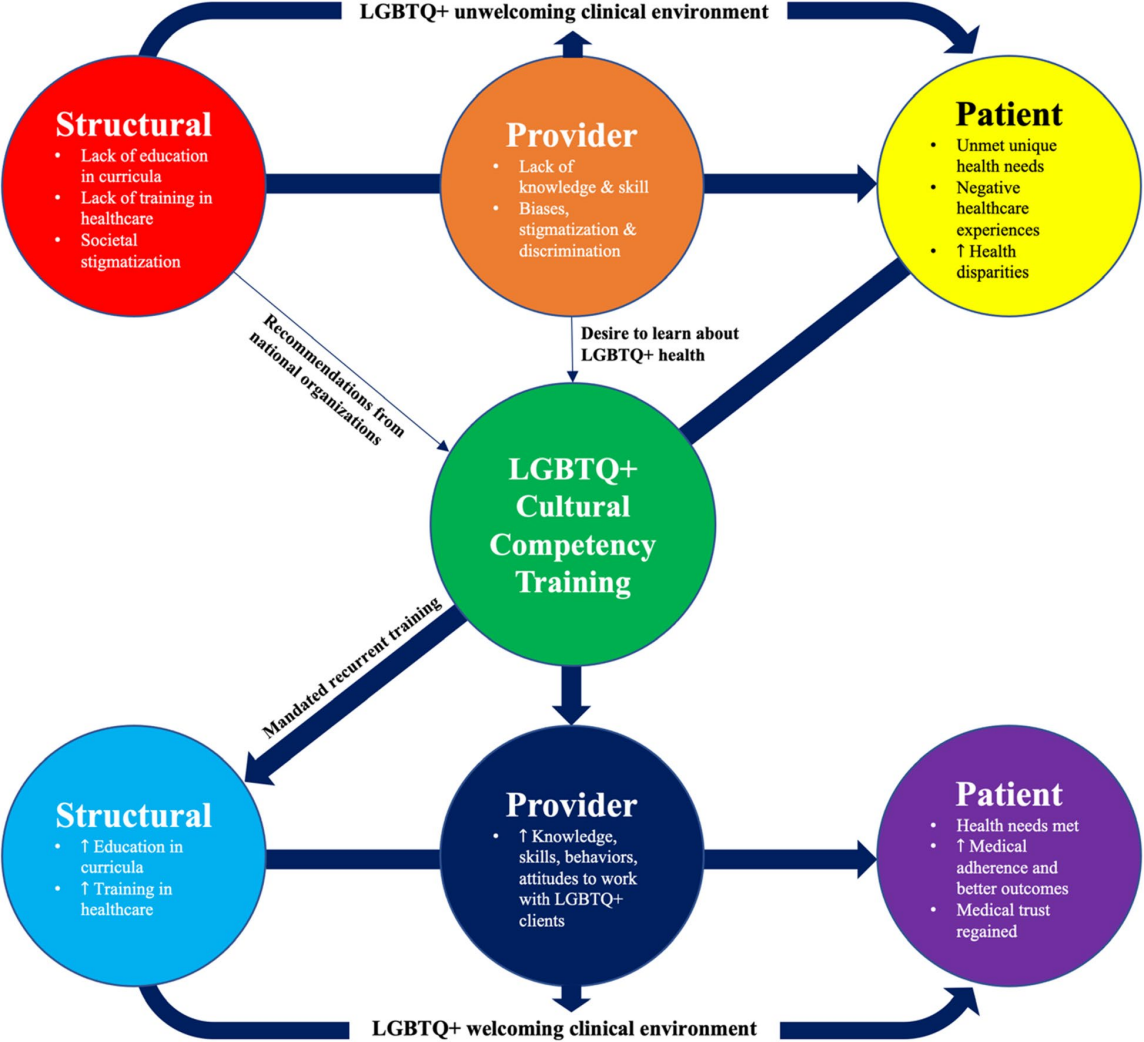
Conceptual Model of LGBTQ + Cultural Competency Training

Health professionals desire more training to address the distinct needs of LGBTQ + individuals, with most concurring that such training must be mandatory [21–24]. However, content and competencies in LGBTQ + health and well-being have not been broadly integrated in health science curricula [25–27], even though national health professional associations, including the American Medical Association [28] and American Nurses Association [29] have advocated for improved training for health professionals to ensure clinically and culturally appropriate care for LGBTQ + patients. The development and provision of LGBTQ + cultural competency training for health professionals have been shaped by a range of multilevel factors, including those at the system, provider, and patient levels, as well as socioecological factors such as laws, policies, and social stigma. A conceptual model of LGBTQ + cultural competency training is presented in Fig. 1.

[Figure 1 shows a conceptual model which reflects three levels of antecedents and consequences for the concept of LGBTQ + cultural competency training: structural-level, provider-level, and patient-level. The three structural antecedents: (1) lack of LGBTQ + health education in curricula, (2) lack of LGBTQ + specific training in healthcare to provide optimal care to LGBTQ + patients, and (3) societal stigmatization of LGBTQ + people, lead to the two provider antecedents: (1) health professionals’ lack of knowledge and skills to work with LGBTQ + clients and (2) their biases, stigmatization and discrimination against LGBTQ + individuals. Also, structural and provider antecedents together create an LGBTQ + unwelcoming clinical environment. These antecedents result in three patient antecedents: (1) LGBTQ + patients’ unmet unique health needs, such as hormone therapies or screenings for anal cancer, (2) negative healthcare experiences, and (3) worse health disparities than their heterosexual and cisgender peers, including medical avoidance due to fear of discrimination and/or medical distrust, which leads to the development and provision of LGBTQ + cultural competency training. As a distinct structural factor, recommendations from national organizations such as the National Institutes of Health, and some health professionals’ desire to learn about LGBTQ + health as a unique provider factor, contribute to the development of the training.

LGBTQ + cultural competency training has three levels of consequences. It increases providers’ cultural competence to work with LGBTQ + patients. Also, as structural consequences of the training, mandated recurrent training within curricula and healthcare institutions further enhances health professionals’ cultural competence. These structural and provider consequences create an LGBTQ + welcoming clinical environment. All these consequences potentially lead to three patient consequences: (1) LGBTQ + patients’ specific health needs are met, (2) they increase medical adherence and improve health outcomes, such as sexual and reproductive health, mental health, cardiovascular and cancer-related outcomes, and (3) the medical trust of LGBTQ + patients may be regained.]

Cultural competence is a complex and multidimensional concept that has evolved over time to meet diverse needs, perspectives, and interests [30, 31]. Several factors have influenced the definitions of cultural competence. These factors include the setting in which the definition is being applied, the cultural background of the individuals involved, the historical and social context in which the definition is developed, and the purpose and intended outcome of the definition [30, 31]. Despite several definitions of cultural competence, the concept has generally been defined as the intricate integration of knowledge, skills, attitudes, and behaviors that improve cross-cultural communication and interpersonal relationships [32–34]. The concept of cultural competence was previously used in the context of race, ethnicity, language, and immigrant or refugee status [35]. More recently, it has been expanded to include sexual orientation and gender identity. Cultural humility, sometimes misused interchangeably with cultural competence, has been described as a lifelong process of learning, self-reflection, and self-critique of the interplay of power, privilege, and social contexts [36, 37].

There has been an ongoing debate on whether cultural competence or cultural humility is a more appropriate value to prioritize by health professionals when interacting with culturally diverse groups. Early models of cultural competence have been criticized due to their focus on competence at the individual level, excluding the structural level, and their implication that a provider can *master* a patient’s lived experiences, and that there is an *end point* where one is sufficiently proficient [37–39]. As a result of these critiques, the concept of cultural competence has evolved to emphasize ongoing engagement instead of terminal training [31].

With cultural competence as a way to support evidence-based practice, LGBTQ + cultural competency training for health professionals aims to enhance their knowledge, skills, attitudes, and behaviors when working with LGBTQ + clients, with the goal of improving patient-provider interactions and leading to better outcomes and satisfaction for LGBTQ + patients [40, 41]. These trainings have been developed and provided to a limited number of health professionals to support the healthcare community’s endeavors to improve healthcare and social service delivery to LGBTQ + patients [41]. These programs have been added as large-scale implementation in some health science curricula and are required by law in at least one jurisdiction (Washington, D.C.) for renewals of licenses for all health professions [4, 42]. Organizational-level policies, which may be necessary to facilitate these trainings, are lacking in healthcare systems, even in those that support and affirm LGBTQ + persons, although such policies were noted as a construct in an original definition of cultural competence [33]. The 2022 report of Healthcare Equality Index [43], which evaluates organizational-level LGBTQ + inclusive policies and practices, notes that even though an increasing number of institutions pursued accreditation, only 55% (496 institutions) met the standard to become LGBTQ + Healthcare Equality Leaders, and they tended to be academic medical centers or located in West and Northeast U.S. regions.

Systematic reviews of LGBTQ + focused trainings have primarily focused on training programs for health professional students [26, 44, 45] or mental health providers [46]. Additionally, none of these reviews have quantitatively evaluated the effectiveness of these programs. While it is important to assess LGBTQ + specific education in health science curricula [25, 26], it is equally crucial to assess the state and effectiveness of post-graduation LGBTQ + cultural competency training programs for health professionals across all disciplines in various healthcare settings.

The strategic plan for 2021–2025 of the National Institutes of Health Sexual & Gender Minority Research Office [47] has underscored the necessity for education about LGBTQ + health and well-being in order for healthcare personnel to provide high quality and individualized care, and to create a welcoming environment for LGBTQ + patients. In response to this call to action and the need for effective LGBTQ + cultural competency training, this systematic review assessed the effectiveness of LGBTQ + cultural competency training programs provided to health professionals across all disciplines in diverse healthcare settings and examined the magnitude of the association between trainings and outcomes, highlighting theory-driven and evidence-based approaches and modalities that may be used for future endeavors to improve the well-being of LGBTQ + individuals.

## Methods

This systematic review complied with the PRISMA (Preferred Reporting Items for Systematic Reviews and Meta-Analyses) statement and checklist [48] to ensure proper reporting of a systematic review. This systematic review was not registered.

### Search strategy

In collaboration with an academic librarian, scientific literature relevant to LGBTQ + cultural competency training among health professionals were collected from five electronic databases: PubMed, CINAHL, PsycINFO, Embase, and Scopus. Search logic was constructed by combining terms associated with *cultural competence, LGBTQ* + *populations*, and *health professionals*. Keywords and/or controlled vocabulary (e.g., Medical Subject Headings [MeSH]) such as “LGBTQ + persons,” “sexual and gender minorities,” “health personnel,” and “cultural competence” and truncations were utilized for each database. Search sets were merged utilizing Boolean operators (AND, OR, NOT). An example of the search strategy used in PubMed is shown in Tables 1 and 2. All references were exported and managed using EndNote 20, with duplicates removed. In addition, manual backward and forward searches were conducted from the identified articles to identify other relevant literature. Manual backward search refers to the process of examining the reference lists of previously identified relevant studies to identify additional studies that may be relevant to the review. Manual forward search involves searching for studies that have cited the previously identified relevant studies [49]. No limit on publication date was set. The search for articles was performed in April 2023.

**Table 1:** Search algorithms

Databases	Search Algorithms
PubMed (323)	(“sexual and gender minorities”[MeSH Terms] OR sexual and gender minorit*[Text Word] OR sexual minorit*[Text Word] OR gender minorit*[Text Word] OR lgbt*[Text Word] OR gay[Text Word] OR lesbian[Text Word] OR bisexual[Text Word] OR transgender[Text Word] OR queer[Text Word] OR intersex[Text Word] OR homosexual*[Text Word]) AND (“health personnel”[MeSH Terms] OR health personnel [Text Word] OR healthcare provider*[Text Word] OR health care provider*[Text Word] OR healthcare professional*[Text Word] OR health care professional*[Text Word] OR healthcare worker*[Text Word] OR health care worker*[Text Word]) AND (“cultural competency”[MeSH Terms] OR cultural competenc*[Text Word] OR culturally competen* [Text Word] OR cultural humility[Text Word] OR culturally humble[Text Word] OR cultural sensitivity*[Text Word] OR culturally sensitive[Text Word] OR cultural responsiveness[Text Word] OR culturally responsive[Text Word]) **Refined by:** English Language
CINAHL (255)	((MH “Sexual and Gender Minorities + ”) OR (MH “LGBTQ + Persons + ”) OR (MH “Gay Persons + ”) OR (MH “Transgender Persons + ”) OR (MM “Gay Men”) OR (MM “Lesbians”) OR (MM “Bisexuals”) OR (MM “Intersex Persons”) OR (MM “Homosexuality”) OR (TX queer) OR (TX “sexual minorit*) OR (TX “gender minorit*)) AND ((MH “Health Personnel + ”) OR (TX healthcare provider*) OR (TX health care provider*) OR (TX healthcare professional*) OR (TX health care professional*) OR (TX healthcare worker*) OR (TX health care worker*)) AND ((MM “Cultural Competence”) OR (TX cultural competenc*) OR (TX culturally competen*) OR (MM “Cultural Sensitivity”) OR (TX culturally sensitive) OR (TX cultural humility) OR (TX culturally humble) OR (TX cultural responsiveness) OR (TX culturally responsive)) **Refined by:** English Language; Academic Journal
PsycINFO (401)	(MAINSUBJECT.EXACT.EXPLODE(“Sexual Minority Groups”) OR MAINSUBJECT.EXACT.EXPLODE(“LGBTQ”) OR MAINSUBJECT.EXACT.EXPLODE(“Bisexuality”) OR MAINSUBJECT.EXACT.EXPLODE(“Transgender”) OR MAINSUBJECT.EXACT.EXPLODE(“Homosexuality”) OR MAINSUBJECT.EXACT.EXPLODE(“Intersex”) OR (queer) OR (gay) OR (lesbian) OR (sexual minorit*) OR (gender minorit*)) AND (MAINSUBJECT.EXACT.EXPLODE(“Health Care Services”) OR (healthcare provider*) OR (health care provider*) OR (healthcare professional*) OR (health care professional*) OR (healthcare worker*) OR (health care worker*)) AND (MAINSUBJECT.EXACT.EXPLODE(“Cultural Competence”) OR MAINSUBJECT.EXACT.EXPLODE(“Cultural Sensitivity”) OR (cultural competenc*) OR (cultural humility) OR (culturally competen*) OR (culturally sensitive) OR (culturally humble) OR (cultural responsiveness) OR (culturally responsive)) **Refined by**: English Language; Peer-reviewed Journal
Embase (511)	((‘sexual and gender minority’/exp OR ‘sexual and gender minorit*’ OR ‘lgbtqia + people’ OR ‘lgbt*’ OR ‘gay’ OR ‘lesbian’ OR ‘transgender’ OR ‘bisexual*’ OR ‘intersex’ OR ‘queer’) AND (‘health care personnel’/exp OR ‘health personnel’ OR ‘healthcare provider*’ OR ‘health care provider*’ OR ‘healthcare professional*’ OR ‘health care professional*’ OR ‘healthcare worker*’ OR ‘health care worker*’) AND (‘cultural competence’/exp OR ‘cultural sensitivity’/exp OR ‘cultural humility’ OR ‘cultural competenc*’ OR ‘culturally competen*’ OR ‘culturally sensitive’ OR ‘culturally humble’ OR ‘cultural responsiveness’ OR ‘culturally responsive’)) **Refined by**: English
Scopus (576)	((TITLE-ABS-KEY (sexual AND gender AND minorit*) OR TITLE-ABS-KEY (sexual AND minorit*) OR TITLE-ABS-KEY (gender AND minorit*) OR TITLE-ABS-KEY (lgbt*) OR TITLE-ABS-KEY (gay) OR TITLE-ABS-KEY (lesbian) OR TITLE-ABS-KEY (bisex*) OR TITLE-ABS-KEY (transgender) OR TITLE-ABS-KEY (queer) OR TITLE-ABS-KEY (intersex) OR TITLE-ABS-KEY (homosexual*))) AND ((TITLE-ABS-KEY (healthcare AND provider*) OR TITLE-ABS-KEY (health AND care AND provider*) OR TITLE-ABS-KEY (healthcare AND professional*) OR TITLE-ABS-KEY (health AND care AND professional*) OR TITLE-ABS-KEY (healthcare AND worker*) OR TITLE-ABS-KEY (health AND care AND worker*) OR TITLE-ABS-KEY (health AND personnel) OR TITLE-ABS-KEY (healthcare AND personnel) OR TITLE-ABS-KEY (health AND care AND personnel))) AND ((TITLE-ABS-KEY (cultural AND competenc*) OR TITLE-ABS-KEY (culturally AND competen*) OR TITLE-ABS-KEY (cultural AND humility) OR TITLE-ABS-KEY (culturally AND humble) OR TITLE-ABS-KEY (cultural AND sensitivity) OR TITLE-ABS-KEY (culturally AND sensitive) OR TITLE-ABS-KEY (cultural responsiveness) OR (culturally responsive))) **Refined by**: English Language; Journal; Article

**Table 2:** PubMed search strategy and results

Search Code	Query	Results
#1	(“sexual and gender minorities”[MeSH Terms] OR sexual and gender minorit*[Text Word] OR sexual minorit*[Text Word] OR gender minorit*[Text Word] OR lgbt*[Text Word] OR gay[Text Word] OR lesbian[Text Word] OR bisexual[Text Word] OR transgender[Text Word] OR queer[Text Word] OR intersex[Text Word] OR homosexual*[Text Word])	58,860
#2	(“health personnel”[MeSH Terms] OR health personnel [Text Word] OR healthcare provider*[Text Word] OR health care provider*[Text Word] OR healthcare professional*[Text Word] OR health care professional*[Text Word] OR healthcare worker*[Text Word] OR health care worker*[Text Word])	729,852
#3	(“cultural competency”[MeSH Terms] OR cultural competenc*[Text Word] OR culturally competen* [Text Word] OR cultural humility[Text Word] OR culturally humble[Text Word] OR cultural sensitivity*[Text Word] OR culturally sensitive[Text Word] OR cultural responsiveness[Text Word] OR culturally responsive[Text Word])	18,183
#4	#1 AND #2 AND #3	323
#5	#1 AND #2 AND #3 Filters: English	323

### Inclusion and exclusion criteria

Articles were included in the review if they: (1) evaluated the effectiveness of LGBTQ + cultural competency trainings, (2) quantitatively measured one or more outcomes of the trainings (e.g., change in knowledge, skills, or attitudes), (3) sampled health professionals in any discipline (e.g., physician, nurse, or social worker), (4) were written in English, and (5) were published in an academic journal. Studies were excluded if they: (1) did not describe their programs as cultural competence or competency training, (2) described a training program without evaluation data, (3) examined the effectiveness of trainings using only qualitative data (e.g., written feedback), (4) did not specifically include topics regarding LGBTQ + populations, and (5) did not engage health professionals (e.g., students only).

Since the level of exposure and experience in providing care to LGBTQ + patients differs between students and working professionals, studies that involved students exclusively were excluded from this review. This was done to achieve the review’s primary aim of assessing the status and effectiveness of LGBTQ + cultural competency training programs provided to health professionals across all disciplines in diverse healthcare settings. To provide a more comprehensive understanding of the impact of LGBTQ + cultural competency training in healthcare settings, training programs engaging non-clinical employees in conjunction with clinical employees were included, given the crucial role that non-clinical employees play in creating an inclusive environment for LGBTQ + patients.

No geographical restrictions were applied in this review. While healthcare systems and medical training models may differ between countries, the experiences and needs of LGBTQ + individuals are not limited to one geographic region. Inclusion of studies from various countries may help identify common themes and best practices in LGBTQ + cultural competency training that can be applied in different healthcare settings around the world.

Two authors (HY/JB) independently screened titles and abstracts for inclusion. Disagreements were resolved via discussion until consensus was met. Full-text articles were assessed for eligibility by the same two authors (HY/JB), and a third author (DF) was consulted for consensus.

### Quality appraisal

To evaluate the quality of quasi-experimental studies, the Joanna Briggs Institute (JBI) critical appraisal checklist for non-randomized experimental studies [50] was used. For the randomized controlled trial study, the JBI checklist for randomized controlled trials [51] was utilized. Each of the appraisal tools consisted of nine or thirteen questions, which were scored using multiple-choice options, including *yes*, *no*, *unclear,* and *not applicable*. To calculate the quality assessment scores, the percentage of questions that were answered “yes” out of the total number of questions (nine or thirteen) for each tool was determined.

The risk of bias of each study was then rated as low (≥ 70%), moderate (50–69%), or high (≤ 49%) [52]. The JBI checklists [50, 51] indicate that studies with a high risk of bias should be investigated further by seeking additional information from the authors, or the study should be excluded. None of the studies had a high risk of bias, thus all studies were included. Quality appraisal results are presented in Tables 3 and 4. To prevent inconsistencies between the initial selection process and the bias assessment process, the author (HY) who selected the articles and another author (DF) who was consulted for consensus were included for the bias assessment process. When disagreements arose, a new reviewer (SB) who was not part of the initial article selection process was involved for consensus to reduce the risk of bias. Prior to consulting with the third author (SB), the agreement rate for the bias assessment was 81.8%.

**Table 3:** Quality assessment results by the JBI critical appraisal checklist for quasi-experimental studies [50]

Author (Year)	Is it clear in the study what is the ‘cause’ and what is the ‘effect’?	Were the participants included in any comparisons similar?	Were the participants included in any comparisons receiving similar treatment or care, other than the exposure or intervention of interest?	Was there a control group?	Were there multiple measurements of the outcome both pre and post the intervention or exposure?	Was follow up complete and if not, were differences between groups in terms of their follow up adequately described and analyzed?	Were the outcomes of participants included in any comparisons measured in the same way?	Were outcomes measured in a reliable way?	Was appropriate statistical analysis used?	Overall quality score
Barrett et al. (2021) [53]	√	√	√	×	×	√	√	√	√	77.8%
Bristol et al. (2018) [54]	√	√	√	×	×	√	√	√	√	77.8%
Craig et al. (2015) [55]	√	?	√	×	×	√	√	√	√	66.7%
Donaldson et al. (2019) [56]	√	√	√	×	×	√	√	√	√	77.8%
Donisi et al. (2020) [57]	√	√	√	×	×	√	√	√	√	77.8%
Felsenstein (2018) [58]	√	√	√	×	×	√	√	√	√	77.8%
Frasca et al. (2019) [59]	√	√	√	×	×	√	√	√	√	77.8%
Gendron et al. (2013) [60]	√	?	√	×	×	√	√	√	√	66.7%
Grova et al. (2021) [61]	√	√	√	×	×	√	√	√	√	77.8%
Hanssmann et al. (2008) [62]	√	?	√	×	×	√	√	√	×	55.6%
Hanssmann et al. (2010) [63]	√	√	√	×	×	√	√	√	√	77.8%
Hardacker et al. (2014) [64]	√	?	√	×	×	×	√	√	√	55.6%
Henry (2017) [65]	√	√	√	×	×	√	√	√	?	66.7%
Holman et al. (2020) [66]	√	√	√	×	×	√	√	√	√	77.8%
Hughes et al. (2016) [67]	√	√	√	×	×	√	√	√	N/A	75%
Ingraham et al. (2016) [68]	√	√	√	×	×	√	√	√	√	77.8%
Jadwin-Cakmak et al. (2020) [69]	√	√	√	×	×	√	√	√	√	77.8%
Kaiafas and Kennedy (2021) [70]	√	√	√	×	×	√	√	√	√	77.8%
Kauth et al. (2016) [71]	√	√	√	×	×	√	√	√	N/A	75%
Kilicaslan and Petrakis (2023) [72]	√	√	√	×	×	√	√	√	N/A	75%
Lelutiu-Weinberger et al. (2016) [73]	√	√	√	×	×	√	√	√	√	77.8%
Leyva et al. (2014) [74]	√	√	√	×	×	√	√	√	√	77.8%
Long et al. (2022) [75]	√	?	√	√	×	√	√	√	√	77.8%
McGarry et al. (2002) [76]	√	√	√	×	×	√	√	√	√	77.8%
Oblea et al. (2022) [77]	√	√	√	×	×	√	√	√	√	77.8%
Pelts and Galambos (2017) [78]	√	√	√	×	×	√	√	√	√	77.8%
Pepping et al. (2018) [79]	√	√	√	×	×	√	√	√	√	77.8%
Pratt-Chapman (2020) [80]	√	√	√	×	×	√	√	√	√	77.8%
Pratt-Chapman (2021) [81]	√	√	√	×	×	√	√	√	√	77.8%
Pratt-Chapman et al. (2022) [82]	√	√	√	×	×	√	√	√	√	77.8%
Rhoten et al. (2021) [83]	√	√	√	×	×	√	√	√	√	77.8%
Rosa-Vega et al. (2020) [84]	√	√	√	×	×	√	√	√	√	77.8%
Russell and Corbitt (2022) [85]	√	√	√	×	×	√	√	√	√	77.8%
Schweiger-Whalen et al. (2019) [86]	√	√	√	×	×	√	√	√	√	77.8%
Seay et al. (2020) [87]	√	√	√	×	×	√	√	√	√	77.8%
Shrader et al. (2017) [88]	√	√	√	×	×	√	√	√	N/A	75%
Stevenson et al. (2020) [89]	√	√	√	×	×	√	√	√	N/A	75%
Traister (2020) [90]	√	√	√	×	×	√	√	√	√	77.8%
Ufomata et al. (2018) [91]	√	√	√	×	×	√	√	√	√	77.8%
Walia et al. (2019) [92]	√	√	√	×	×	√	√	√	√	77.8%
Weeks et al. (2018) [93]	√	√	√	×	×	√	√	√	√	77.8%
White-Hughto et al. (2017) [94]	√	√	√	×	×	√	√	√	√	77.8%
Wyckoff (2019) [95]	√	√	√	×	×	√	√	√	√	77.8%

**Table 4:** Quality assessment results by the JBI critical appraisal checklist for randomized controlled trials [51]

Author (Year)	Was true randomization used for assignment of participants to treatment groups?	Was allocation to treatment groups concealed?	Were treatment groups similar at the baseline?	Were participants blind to treatment assignment?	Were those delivering treatment blind to treatment assignment?	. Were outcomes assessors blind to treatment assignment?	Were treatment groups treated identically other than the intervention of interest?	Was follow up complete and if not, were differences between groups in terms of their follow up adequately described and analyzed?	Were participants analyzed in the groups to which they were randomized?	Were outcomes measured in the same way for treatment groups?	Were outcomes measured in a reliable way?	Was appropriate statistical analysis used?	Was the trial design appropriate, and any deviations from the standard RCT design (individual randomization, parallel groups) accounted for in the conduct and analysis of the trial?	Overall quality score
Pachankis et al. (2022) [96]	√	√	√	?	?	?	√	√	√	√	√	√	√	76.9%

^*^√ = Yes, ×  = No, ? = Unclear, *N/A* Not applicable

### Data extraction

Data from each individual study were extracted and compiled into a table of evidence through the matrix method [97]. The extracted data captured critical information on the following: the study design, country/region, sample characteristic, training setting (e.g., voluntary or mandatory), theoretical framework, source of training material, training topic, training modality, duration, trainer, training target, measurement instrument, effect size and key findings.

### Data synthesis

After collecting the data, two authors (HY/SB) used an abductive approach to analyze and synthesize the data. This approach combines inductive and deductive methods to gain a comprehensive understanding of the subject under study [98]. First, an inductive approach was used to identify the key characteristics of the studies, samples, and training programs that may affect the effectiveness of these program. This involved analyzing similarities and differences in these characteristics across included studies, including the use of LGBTQ + trainers, longer training durations, and voluntary settings. The data was categorized using the major steps of content analysis, including decontextualization, recontextualization, categorization, and compilation of data [99]. Once potential patterns or themes that may influence the effectiveness of the programs were identified, the primary author (HY) analyzed measurement items in the main text and supplementary section of each study to synthesize the training outcomes. A total of 264 measurement items were reviewed thoroughly to create outcome categories, and the second author (SB) was consulted for discussion when there was a discrepancy between a measurement item and the study’s stated target outcome. Finally, a deductive approach was used to examine the relationships between the identified study, sample, and training characteristics and the synthesized outcomes. This involved comparing specific training outcomes based on whether the identified characteristics were present.

To understand the magnitude of the association between trainings and outcomes, effect sizes were calculated as a quantitative measure and reported as Hedges’ *g* after correcting for bias from Cohen’s *d*, which was computed from the output of *t*-tests, or omega squared (ω^2^) after correcting for bias from eta squared (η^2^), which was computed from the output of an analysis of variance [100]. Based upon benchmarks [101], Hedges’ *g* was rated as large (≥ 0.8), medium (0.5–0.79), small (0.2–0.49), or trivial (< 0.2); omega squared (ω^2^) was graded as large (≥ 0.14), medium (0.06–0.13), small (0.01–0.05), or trivial (< 0.01). Effect sizes were calculated using Microsoft Excel and are presented in Table 6.

## Results

**Fig. 2:**
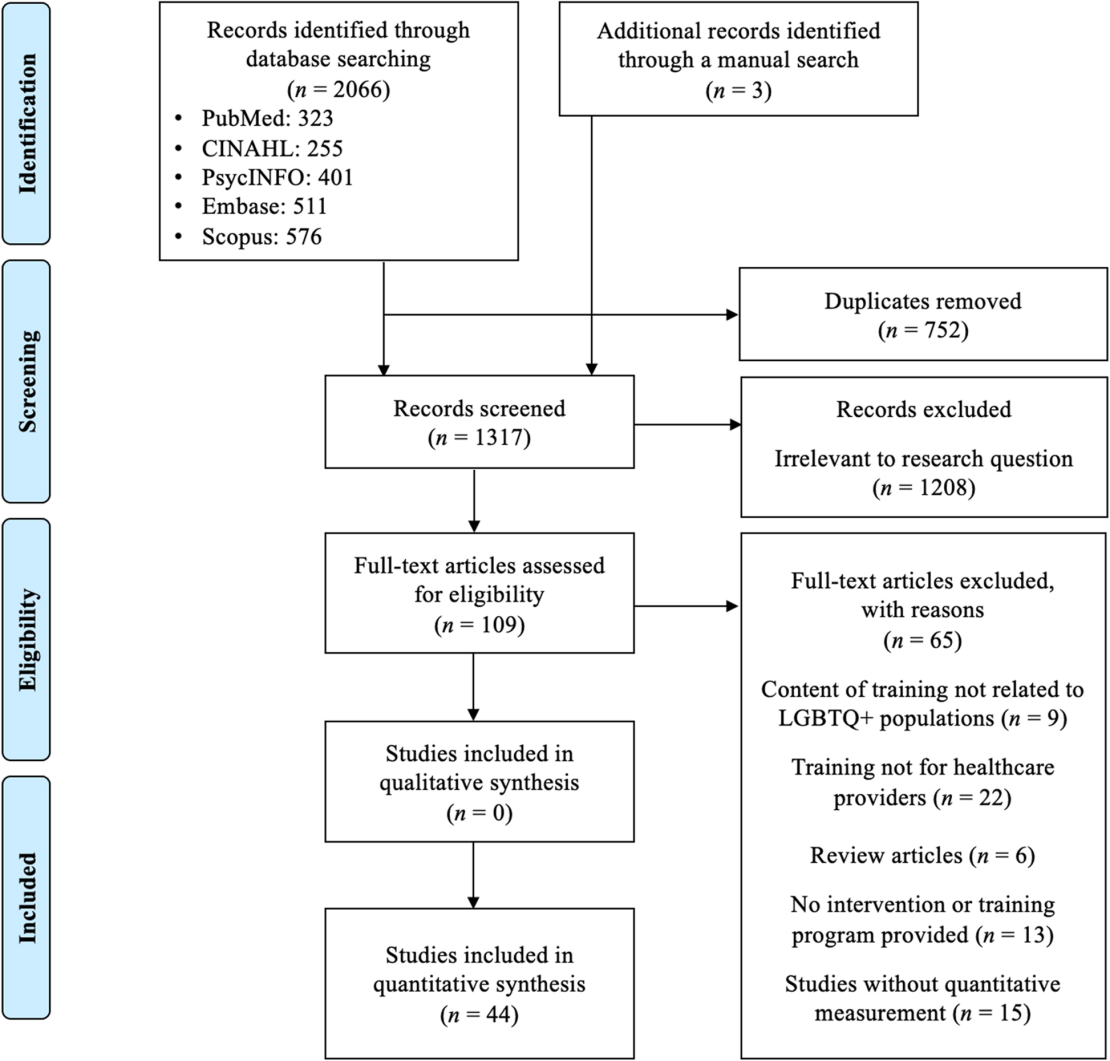
PRISMA Flow Diagram

A total of 2,069 citations were identified from the five electronic databases. After duplicates were removed, 1,317 unique abstracts remained. An additional 1,208 abstracts were excluded in the title and abstract screening phase due to their irrelevance to the aim of the review. We retrieved 109 studies for full text review. 65 articles were excluded in the full text screening phase, because: (1) trainings were not related to LGBTQ + populations (*n* = 9), (2) trainings did not engage health professionals (*n* = 22), (3) studies were review articles (*n* = 6), (4) studies did not provide training programs (*n* = 13), and (5) studies did not employ quantitative measurements (*n* = 15). The remaining 41 articles and three additional articles identified from a manual backward and forward search met inclusion criteria, so a total of 44 studies were included. A flow diagram [102] of the literature search is presented in Fig. 2.

[Figure 2 displays the flow of study identification and selection. The initial search of the database yielded 2066 records, comprising 323 records from PubMed, 255 records from CINAHL, 401 records from PsycINFO, 511 records from Embase, and 576 records from Scopus. Three additional records were identified from manual forward and backward searches. After removing duplicates, 1317 unique citations were subjected to title and abstract screening. In the first screening phase, 1208 records were excluded for being irrelevant to our review aims, leaving 109 records for full-text screening. In the second screening phase, 65 articles were excluded for various reasons: 9 did not pertain to an LGBTQ + population, 22 did not involve health professionals, 6 were review articles, 13 did not offer training programs, and 15 did not evaluate training programs quantitatively. Forty-four articles were included in the quantitative synthesis.]

### Study characteristics

A summary of the 44 studies reviewed can be found in Table 5. The included studies were conducted in various countries, including the United States (*n* = 39), Canada (*n* = 1), Australia (*n* = 2), Europe (*n* = 1), and a mixed group of countries, including the U.S., Canada, and Uganda (*n* = 1). Among the 39 U.S. studies, studies were conducted in several regions as defined by United States Census Bureau [103]: West (*n* = 10), Midwest (*n* = 8), South (*n* = 11), Northeast (*n* = 6), Hawaii (*n* = 1), Puerto Rico (*n* = 1), and mixed regions (*n* = 2).

**Table 5:** Population and training characteristics across studies

Author (Year)	Country (Region)	Study Design	Sample Characteristic/Setting	Training Topic	Theoretical Framework	Source of Training Material	Training Modality (Program Format)	Duration	Trainer
Barrett et al. (2021) [53]	USA (Georgia)	Quasi-experimental, pretest–posttest without control	Training type: voluntary Setting: primary care (dermatology clinic) Clinical staff: dermatology resident, medical student (*N* = 29)	Population focus: all LGBTQ + populations Topics: distinct health needs and care considerations for LGBTQ + individuals, health disparities for LGBTQ + persons, strategies to create a welcoming and inclusive environment for LGBTQ + clients, unique lived experiences of LGBTQ + persons (pre-recorded video)			Approach: Multimodal Training mode: online (Didactic lecture, video, role-play simulation)	2 h	Lecturer and facilitator (Not specified)
Bristol et al. (2018) [54]	USA (Maryland)	Quasi-experimental, pretest–posttest without control (Posttest occurred 3 to 5 months after training)	Training type: voluntary Setting: acute care (emergency department) Clinical staff: nurse, nurse practitioner, physician Non-clinical staff: unit secretary (*N* = 135)	Population focus: all LGBTQ + populations Topics: LGBTQ + terminology and culture, health disparities for LGBTQ + persons, strategies to create a welcoming and inclusive environment for LGBTQ + clients, intersectionality		National LGBT Cancer Network	Approach: Multimodal Training mode: mixed online lecture and in-person activities (Didactic lecture using online module, interactive exercises, group activities, and short films)	6 h	Nurse and nurse educator in the emergency department
Craig et al. (2015) [55]	Canada	Quasi-experimental, posttest only without control (Data collected with recurrent interventions over 2.5 years)	Training type: voluntary Setting: mixed healthcare settings (not specified) Clinical staff: nurse, social worker, psychologist Non-clinical staff: teacher, professor, sales associate, lawyer (*N* = 2850, randomly selected from 8550 samples)	Population focus: LGB youth Topics: LGBTQ + terminology and culture, health disparities for LGBTQ + persons, LGBTQ + inclusive clinical practice knowledge and skills	Information-Motivation-Behavioral Skills model		Approach: Multimodal Training mode: in-person (Didactic lecture, group discussion)	1–3 h	Trainers from community organizations
Donaldson et al. (2019) [56]	USA (Wisconsin)	Quasi-experimental, pretest–posttest without control	Training type: voluntary Setting: long-term care Clinical staff: nurse, physician, social worker, occupational therapist, physical therapist, psychologist Non-clinical staff: recreation therapist, administrative staff, chaplain (N = 26)	Population focus: LGBTQ + veterans Topics: LGBTQ + terminology and culture, intersectionality, health disparities for LGBTQ + persons, distinct health needs and care considerations for LGBTQ + individuals			Approach: Single modality Training mode: online (Didactic lecture using online module)	1 h	
Donisi et al. (2020) [57]	Belgium, Italy, Poland, UK	Quasi-experimental, pretest–posttest without control	Training type: voluntary Setting: mixed healthcare settings (not specified) Clinical staff: physician, nurse, psychologist Non-clinical staff: support staff (*N* = 102)	Population focus: all LGBTQ + populations Topics: LGBTQ + terminology and culture, health disparities for LGBTQ + persons, LGBTQ + inclusive clinical practice knowledge and skills, distinct health needs and care considerations for LGBTQ + individuals			Approach: Multimodal Training mode: in-person (Didactic lecture, small group activities, large group discussion, role-play simulation, case studies, videos, reflective practice)		Healthcare professionals & LGBTQ + trainer
Felsenstein (2018) [58]	USA (Minnesota)	Quasi-experimental, pretest–posttest without control	Training type: mandatory Setting: primary care clinic Clinical staff: healthcare professionals (Not specified) Non-clinical staff: administrative staff (*N* = 11)	Population focus: all LGBTQ + populations Topics: LGBTQ + terminology and culture, distinct health needs and care considerations for LGBTQ + individuals	Change theory	GLMA: Health Professionals Advancing LGBTQ Equality	Approach: Multimodal Training mode: mixed online lecture and in-person activities (Didactic lecture using online module and panel presentation from professionals who provide LGBTQ + focused care)	2.5 h	Facilitators and healthcare professionals
Frasca et al. (2019) [59]	USA (Colorado)	Quasi-experimental, pretest–posttest without control	Training type: mandatory Setting: primary care (HIV clinic) Clinical staff: internal medicine resident (N = 19)	Population focus: all LGBTQ + populations Topics: LGBTQ + terminology and culture, LGBTQ + inclusive clinical practice knowledge and skills, health disparities for LGBTQ + persons, distinct health needs and care considerations for LGBTQ + individuals	The model of four levels of training evaluation	National LGBTQIA + Health Education Center, GLMA: Health Professionals Advancing LGBTQ Equality	Approach: Single modality Training mode: online (Patient case-based online module)	8 h	
Gendron et al. (2013) [60]	USA (Virginia)	Quasi-experimental, pretest–posttest without control	Training type: mixed mandatory and voluntary Setting: mixed healthcare settings (not specified) Clinical staff: healthcare professionals (Not specified) (*N* = 158)	Population focus: LGBTQ + older adults Topics: LGBTQ + terminology and culture, LGBTQ + inclusive clinical practice knowledge and skills, unique lived experiences of LGBTQ + persons			Approach: Multimodal Training mode: in-person (Didactic lecture, interactive exercises, small group activities, Gen Silent documentary, role-play simulation)	2 h versus 4 h	Trainers and facilitators (not specified)
Grova et al. (2021) [61]	USA (North Carolina)	Quasi-experimental, pretest–posttest without control (Posttest occurred 6 weeks after training)	Training type: voluntary Setting: acute care (academic medical center) Clinical staff: surgical residents (*N* = 28)	Population focus: all LGBTQ + populations Topics: LGBTQ + terminology and culture, health disparities for LGBTQ + persons, LGBTQ + inclusive clinical practice knowledge and skills		National LGBTQIA + Health Education Center	Approach: Single modality Training mode: in-person (Didactic lecture)	2 h	Facilitator (not specified)
Hanssmann et al. (2008) [62]	USA (Washington)	Quasi-experimental, pretest–posttest without control (Data collected with recurrent interventions over 2.5 years)	Training type: voluntary Setting: mixed healthcare settings (not specified) Clinical staff: healthcare professionals (Not specified) (*N* = 55)	Population focus: transgender and gender-nonconforming patients Topics: LGBTQ + terminology and culture, health disparities for LGBTQ + persons, distinct health needs and care considerations for LGBTQ + individuals, unique lived experiences of LGBTQ + persons			Approach: Multimodal Training mode: in-person (Didactic lecture, LGBTQ + panel presentation and group discussion)	1 h to a full day	Trainers from a community organization, including a transgender trainer and transgender youth panelist
Hanssmann et al. (2010) [63]	USA (Washington)	Quasi-experimental, pretest–posttest without control	Training type: voluntary Setting: mixed healthcare settings (community hospital, community health clinic) Clinical staff: physician, nurse practitioner, nurse, medical assistant, health educator, school counselor, prevention specialist (*N* = 55)	Population focus: transgender and gender-nonconforming patients Topics: LGBTQ + terminology and culture, distinct health needs and care considerations for LGBTQ + individuals, health disparities for LGBTQ + persons, unique lived experiences of LGBTQ + persons			Approach: Multimodal Training mode: in-person (Didactic lecture, LGBTQ + panel presentation and interactive exercises)	1 h to 6 h	Trainers from a community organization, including a transgender trainer and transgender youth panelist
Hardacker et al. (2014) [64]	USA (Illinois)	Quasi-experimental, pretest–posttest without control (Data collected with recurrent interventions over 2.5 years)	Training type: mandatory Setting: mixed healthcare settings (academic setting, community-based health center, home health-care network, nursing home) Clinical staff: nurse, nursing assistant, physician, medical student (N = 848)	Population focus: LGBTQ + older adults Topics: LGBTQ + terminology and culture, structural and systemic oppression of LGBTQ + people, health disparities for LGBTQ + persons, distinct health needs and care considerations for LGBTQ + individuals, LGBTQ + inclusive clinical practice knowledge and skills, strategies to create a welcoming and inclusive environment for LGBTQ + clients			Approach: Multimodal Training mode: in-person (Didactic lecture, group discussion, practical activity)	6 h	Facilitator (not specified)
Henry (2017) [65]	USA (Delaware)	Quasi-experimental, pretest–posttest without control	Training type: voluntary Setting: primary care (psychiatric clinic) Clinical staff: clinical nurse specialist, nurse, nurse practitioner, social worker Non-clinical staff: front office staff (*N* = 8)	Population focus: all LGBTQ + populations Topics: health disparities for LGBTQ + persons, unique lived experiences of LGBTQ + persons	Theory of interpersonal relations		Approach: Multimodal Training mode: in-person (Didactic lecture, LGBTQ + panel presentation, video, group discussion)		Researcher & LGBTQ + panelists
Holman et al. (2020) [66]	USA (Ohio)	Quasi-experimental, pretest–posttest without control	Training type: mandatory Setting: community (senior living facility) Clinical staff: nurse Non-clinical staff: social support, activity director, janitorial crew, kitchen staff, dietician, and administrative assistant) at senior living facility (*N* = 43)	Population focus: LGBTQ + older adults Topics: LGBTQ + terminology and culture, structural and systemic oppression of LGBTQ + people, distinct health needs and care considerations for LGBTQ + individuals, LGBTQ + inclusive clinical practice knowledge and skills	Minority stress theory	National Resource Center on LGBTQ + Aging	Approach: Multimodal Training mode: in-person (Didactic lecture, role-play simulation, small group activities)	4 h	Trainers and facilitators (Not specified)
Hughes et al. (2016) [67]	USA (Michigan)	Quasi-experimental, pretest–posttest without control	Training type: voluntary Setting: mixed healthcare settings (not specified) Clinical staff: health professional in the aging services network and in long term care, public mental health provider Non-clinical staff: public health administrative staff (*N* = 204)	Population focus: LGBTQ + older adults Topics: health disparities for LGBTQ + persons			Approach: Multimodal Training mode: in-person (Transformative theatre experience, including theatrical performance, interactive dialogue, small group work, and larger group discussion)	2 h	Actors (LGBTQ + and non-LGBTQ + persons), facilitator
Ingraham et al. (2016) [68]	USA (California)	Quasi-experimental, pretest–posttest without control (Academic format and clinic format groups)	Training type: voluntary Setting: mixed healthcare settings (community health center, university) Clinical staff: physician, resident, nurse, medical assistant, nurse practitioner Non-clinical staff: front desk staff (*N* = 92)	Population focus: Lesbian and bisexual women (LB) Topics: LGBTQ + terminology and culture, health disparities for LGBTQ + persons	Theory of cultural competence in healthcare delivery and the concept of motivational interviewing		Approach: Multimodal Training mode: in-person (Didactic lecture, skill development exercises, role-play simulation)	1 h to 3 h	Program staff (not specified)
Jadwin-Cakmak et al. (2020) [69]	USA (Michigan)	Quasi-experimental, pretest–posttest without control (Posttest occurred 6 months after training)	Training type: voluntary Setting: primary care (community health center, pediatric clinic) Clinical staff: physician, physician assistant, nurse practitioner, nurse, medical assistant, nursing assistant, social worker, psychologist, counselor, health educator, community health worker Non-clinical staff: administrator, medical office clerk, executive director, chief executive officer (*N* = 153)	Population focus: LGBTQ + youth Topics: LGBTQ + terminology and culture, bias assessment and mitigation, distinct health needs and care considerations for LGBTQ + individuals, LGBTQ + inclusive clinical practice knowledge and skills	Situated Information-Motivation-Behavioral Skills Model of Care Initiation and Maintenance		Approach: Multimodal Training mode: mixed online lectures and in-person activities (Didactic lecture, group activities, role-play simulation)	3 h	Medical doctor, health educator
Kaiafas and Kennedy (2021) [70]	USA (North Carolina)	Quasi-experimental, pretest–posttest without control	Training type: voluntary Setting: acute care (emergency department) within the military health system Clinical staff: nurse (*N* = 72)	Population focus: all LGBTQ + populations Topics: LGBTQ + terminology and culture, health disparities for LGBTQ + persons, LGBTQ + inclusive clinical practice knowledge and skills, distinct health needs and care considerations for LGBTQ + individuals		National LGBTQIA + Health Education Center	Approach: Multimodal Training mode: in-person (Didactic lecture, open discussion, small group practice scenarios)	2.5 h	Educator in the emergency department
Kauth et al. (2016) [71]	USA (Washington, DC)	Quasi-experimental, posttest only without control (Data collected with recurrent interventions over 2 years)	Training type: mandatory Setting: mixed healthcare settings within the Veterans Health Administration Clinical staff: clinical psychologist in interprofessional postdoctoral psychology fellowships in LGBTQ + health (*N* = 16)	Population focus: LGBTQ + veterans Topics: distinct health needs and care considerations for LGBTQ + individuals, health disparities for LGBTQ + persons	The framework of interprofessional collaborative practice		Approach: Multimodal Training mode: in-person (Didactic lecture, training/teaching others, research)		Mentor and supervisors
Kilicaslan and Petrakis (2023) [72]	Australia	Quasi-experimental, posttest only without control	Training type: voluntary Setting: mixed healthcare settings within the public hospital clinical mental health service Clinical staff: nurse, social worker, occupational therapist, psychiatrist, psychologist Non-clinical staff: lived experience worker (*N* = 113)	Population focus: all LGBTQ + populations Topics: LGBTQ + terminology and culture, health disparities for LGBTQ + persons, structural and systemic oppression of LGBTQ + people, LGBTQ + inclusive clinical practice knowledge and skills	Minority stress theory		Approach: Multimodal Training mode: in-person (Didactic lecture, video, group discussion)	1 h	Mental health staff (psychiatric nurse, medical psychiatry registrar, occupational therapist, consumer peer support worker)
Lelutiu-Weinberger et al. (2016) [73]	USA (New York)	Quasi-experimental, pretest–posttest without control (Posttest occurred 3 months after training)	Training type: mandatory Setting: primary care (outpatient clinic) Clinical staff: physician, nurse, prevention counselor, social service provider, patient coordinator Non-clinical staff: registrar, administrative staff, security guard, billing staff (*N* = 32)	Population focus: transgender patients Topics: LGBTQ + terminology and culture, distinct health needs and care considerations for LGBTQ + individuals, health disparities for LGBTQ + persons, strategies to create a welcoming and inclusive environment for LGBTQ + clients, LGBTQ + inclusive clinical practice knowledge and skills			Approach: Multimodal Training mode: in-person (Didactic lecture, practice-based example scenarios, group discussion)	6 h	Nurse practitioner and physician
Leyva et al. (2014) [74]	USA (California)	Quasi-experimental, pretest–posttest without control	Training type: voluntary Setting: mixed healthcare settings (not specified) Clinical staff: social worker, counselor, nurse, first responder, skilled nursing and other residential care facility manager and staff member Non-clinical staff: senior services ombudsmen, religious leader (*N* = 115)	Population focus: LGBTQ + older adults Topics: LGBTQ + terminology and culture, strategies to create a welcoming and inclusive environment for LGBTQ + clients, structural and systemic oppression of LGBTQ + people, unique lived experiences of LGBTQ + persons			Approach: Multimodal Training mode: in-person (Didactic lecture, group discussion, LGBTQ + panel presentation)	1 day	Experts and LGBTQ + older adult panelists
Long et al. (2022) [75]	USA (Maryland)	Quasi-experimental, posttest only with control (the control group did not attend the event) (Data collected with recurrent interventions over 2 years)	Training type: voluntary Setting: mixed healthcare settings (not specified) Clinical staff: physician, nurse, social worker, case manager Non-clinical staff: administrative staff (*N* = 111)	Population focus: all LGBTQ + populations Topics: unique lived experiences of LGBTQ + persons, health disparities for LGBTQ + persons			Approach: Single modality Training mode: in-person (Storytelling event: healthcare providers and LGBTQ + persons share their own stories)	1.5 h	Event facilitator and LGBTQ + storytellers
McGarry et al. (2002) [76]	USA (Rhode Island)	Quasi-experimental, pretest–posttest without control	Training type: mandatory Setting: mixed healthcare settings (not specified) Clinical staff: physician (internal medicine resident) (N = 37)	Population focus: lesbian and gay patients Topics: health disparities for LGBTQ + persons, distinct health needs and care considerations for LGBTQ + individuals, LGBTQ + inclusive clinical practice knowledge and skills			Approach: Multimodal Training mode: in-person (Didactic lecture, video, case discussion)	3 h	Faculty
Oblea et al. (2022) [77]	USA (Hawaii)	Quasi-experimental, pretest–posttest without control	Training type: voluntary Setting: mixed healthcare settings (not specified) in both civilian and military health systems Clinical staff: physician, psychologist, nurse, social worker, counselor, case manager, dentist, first responder Non-clinical staff: administrator (*N* = 101)	Population focus: all LGBTQ + populations Topics: LGBTQ + terminology and culture, health disparities for LGBTQ + persons, unique lived experiences of LGBTQ + persons, strategies to create a welcoming and inclusive environment for LGBTQ + clients, LGBTQ + inclusive clinical practice knowledge and skills			Approach: Multimodal Training mode: in-person (Didactic lecture, video, interactive activity, case discussion)	4 h	Facilitator (university faculty) and a transgender panel
Pachankis et al. (2022) [96]	USA, Canada, Uganda	Randomized controlled trial	Training type: voluntary Setting: mixed healthcare settings (not specified) Clinical staff: licensed or unlicensed mental health provider (*N* = 121)	Population focus: all LGBTQ + populations Topics: structural and systemic oppression of LGBTQ + people, distinct health needs and care considerations for LGBTQ + individuals, intersectionality, LGBTQ + inclusive clinical practice knowledge and skills, health disparities for LGBTQ + persons	Minority stress theory		Approach: Multimodal Training mode: online (Didactic lectures, video-based simulated practice assessment)	11 h	Licensed clinical psychologists and a counseling psychologist who self-identify as LGBTQ +
Pelts and Galambos (2017) [78]	USA (Missouri)	Quasi-experimental, pretest–posttest without control	Training type: voluntary Setting: long-term care Clinical staff: nurse, social worker, nursing assistant Non-clinical staff: activities or other support staff (*N* = 42)	Population focus: lesbian and gay older adults Topics: health disparities for LGBTQ + persons, unique lived experiences of LGBTQ + persons, structural and systemic oppression of LGBTQ + people	Intergroup contact (IGC) theory		Approach: Multimodal Training mode: in-person (Video, group discussion)		Facilitator
Pepping et al. (2018) [79]	Australia	Quasi-experimental, pretest–posttest without control	Training type: voluntary Setting: mixed healthcare settings (not specified) Clinical staff: mental health professional (licensed psychologist, social worker, psychiatrist, medical practitioner, mental health practitioner) (*N* = 96)	Population focus: all LGBTQ + populations Topics: LGBTQ + terminology and culture, health disparities for LGBTQ + persons, LGBTQ + inclusive clinical practice knowledge and skills, distinct health needs and care considerations for LGBTQ + individuals, unique lived experiences of LGBTQ + persons	Minority stress theory		Approach: Multimodal Training mode: in-person (Didactic lecture, video, group discussion, reflection exercises)	7.5 h	Clinical psychologist
Pratt-Chapman (2020) [80]	USA (American Samoa, California, Florida, Hawaii, Louisiana, Maine, Massachusetts, Michigan, Minnesota, and Montana)	Quasi-experimental, pretest–posttest without control	Training type: voluntary Setting: mixed healthcare settings (not specified) Clinical staff: physician, nurse, nurse practitioner, social worker Non-clinical staff: administrator (*N* = 28)	Population focus: all LGBTQ + populations Topics: distinct health needs and care considerations for LGBTQ + individuals, strategies to create a welcoming and inclusive environment for LGBTQ + clients			Approach: Multimodal Training mode: mixed online lecture and in-person activities (Didactic lecture using online module, in-person workshop)	8.7 h	
Pratt-Chapman (2021) [81]	USA (Arizona)	Quasi-experimental, pretest–posttest without control	Training type: voluntary Setting: mixed healthcare settings (not specified) Clinical staff: social worker (*N* = 26)	Population focus: all LGBTQ + populations Topics: LGBTQ + terminology and culture, intersectionality, structural and systemic oppression of LGBTQ + people, health disparities for LGBTQ + persons, LGBTQ + inclusive clinical practice knowledge and skills, unique lived experiences of LGBTQ + persons		National LGBT Cancer Network	Approach: Multimodal Training mode: in-person (Didactic lecture, small group discussion, role-play simulation, case vignettes, films)	3 h	
Pratt-Chapman et al. (2022) [82]	USA (Six states, not specified)	Quasi-experimental, pretest–posttest without control	Training type: voluntary Setting: mixed healthcare settings (cancer centers, cancer screening program, community-based organization) Clinical staff: physician, nurse, nurse practitioner, community health worker, social worker Non-clinical staff: non-clinical role (not specified) (*N* = 22)	Population focus: all LGBTQ + populations Topics: health disparities for LGBTQ + persons, intersectionality, structural and systemic oppression of LGBTQ + people, LGBTQ + inclusive clinical practice knowledge and skills, strategies to create a welcoming and inclusive environment for LGBTQ + clients, bias assessment and mitigation	Fundamental cause theory and intersectionality		Approach: Multimodal Training mode: online (Didactic lecture using online module, virtual interactive session, including information session and activities)	14.5 h	Researcher
Rhoten et al. (2021) [83]	USA (New York)	Quasi-experimental, pretest–posttest without control (Data collected with recurrent interventions over 5 years)	Training type: voluntary Setting: mixed healthcare settings (hospital, clinic) Clinical staff: hospital staff and primary care provider (*N* = 420)	Population focus: all LGBTQ + populations Topics: LGBTQ + terminology and culture, health disparities for LGBTQ + persons, structural and systemic oppression of LGBTQ + people, intersectionality, distinct health needs and care considerations for LGBTQ + individuals, strategies to create a welcoming and inclusive environment for LGBTQ + clients	Adult learning theory and transformative learning theory	National LGBT Cancer Network	Approach: Multimodal Training mode: in-person (Didactic lecture, activities, discussion, video)		Certified trainers (not specified)
Rosa-Vega et al. (2020) [84]	USA (Puerto Rico)	Quasi-experimental, pretest–posttest without control	Training type: voluntary Setting: mixed healthcare settings (hospital pharmacy, community pharmacy, pharmaceutical industry) Clinical staff: pharmacist (*N* = 54)	Population focus: transgender patients Topics: LGBTQ + terminology and culture, health disparities for LGBTQ + persons, distinct health needs and care considerations for LGBTQ + individuals			Approach: Multimodal Training mode: in-person (Didactic lecture, case discussion)	3 h	Panel of presenters (not specified)
Russell and Corbitt (2022) [85]	USA (Maryland)	Quasi-experimental, pretest–posttest without control	Training type: voluntary Setting: acute care (academic cancer center) Clinical staff: nurse, medical assistant, radiation therapy technician, patient care technician, social worker, physician, nurse practitioner, physician assistant Non-clinical staff: clerical staff, administrative staff (*N* = 110)	Population focus: all LGBTQ + populations Topics: LGBTQ + terminology and culture, health disparities for LGBTQ + persons, structural and systemic oppression of LGBTQ + people, intersectionality, distinct health needs and care considerations for LGBTQ + individuals, bias assessment and mitigation, strategies to create a welcoming and inclusive environment for LGBTQ + clients, unique lived experiences of LGBTQ + persons	Minority stress theory and intersectionality	National LGBT Cancer Network	Approach: Multimodal Training mode: in-person (Didactic lecture, interactive exercises, group discussion, video)	1 h	Trained healthcare professionals
Schweiger-Whalen et al. (2019) [86]	USA (New Mexico)	Quasi-experimental, pretest–posttest without control	Training type: voluntary Setting: mixed healthcare settings (hospital, university) Clinical staff: nurse, nursing student, nurse practitioner, social worker, counselor, physical therapist, pharmacist Non-clinical staff: nursing student, nursing faculty, administrative (*N* = 130)	Population focus: all LGBTQ + populations Topics: LGBTQ + terminology and culture, health disparities for LGBTQ + persons, LGBTQ + inclusive clinical practice knowledge and skills, unique lived experiences of LGBTQ + persons	Theory of cultural competence in healthcare delivery	National LGBTQIA + Health Education Center	Approach: Multimodal Training mode: in-person (Didactic lecture, LGBTQ + panel presentation)	4 h	Educator and LGBTQ + panelists
Seay et al. (2020) [87]	USA (Florida)	Quasi-experimental, pretest–posttest without control	Training type: voluntary Setting: acute care (academic cancer center) Clinical staff: oncologist (*N* = 40)	Population focus: all LGBTQ + populations Topics: LGBTQ + terminology and culture, intersectionality, strategies to create a welcoming and inclusive environment for LGBTQ + clients, LGBTQ + inclusive clinical practice knowledge and skills, health disparities for LGBTQ + persons	Theory of cultural competence in healthcare delivery	National LGBT Cancer Network	Approach: Single modality Training mode: online (Didactic lecture using online module)	2 h	
Shrader et al. (2017) [88]	USA (California, Washington)	Quasi-experimental, pretest–posttest without control	Training type: voluntary Setting: primary care within the military health system Clinical staff: psychologist, psychiatrist, social worker, nurse practitioner, physician (*N* = 51)	Population focus: all LGBTQ + populations Topics: LGBTQ + terminology and culture, structural and systemic oppression of LGBTQ + people, distinct health needs and care considerations for LGBTQ + individuals, health disparities for LGBTQ + persons		National LGBTQIA + Health Education Center	Approach: Single modality Training mode: in-person (Didactic lecture)	1 h	Researcher
Stevenson et al. (2020) [89]	USA (Georgia)	Quasi-experimental, pretest–posttest without control	Training type: mandatory Setting: primary care (endocrinology) Clinical staff: endocrine fellow (*N* = 6)	Population focus: transgender patients Topics: distinct health needs and care considerations for LGBTQ + individuals, LGBTQ + inclusive clinical practice knowledge and skills			Approach: Single modality Training mode: in-person (Simulation using a standardized patient)		Faculty
Traister (2020) [90]	USA (Pennsylvania)	Quasi-experimental, pretest–posttest without control	Training type: voluntary Setting: acute care (hospital) Clinical staff: nurse (*N* = 112)	Population focus: all LGBTQ + populations Topics: LGBTQ + terminology and culture, health disparities for LGBTQ + persons, LGBTQ + inclusive clinical practice knowledge and skills	Theory of cultural competence in healthcare delivery	National LGBTQIA + Health Education Center	Approach: Single modality Training mode: in-person (Didactic lecture)	1 h	Researcher
Ufomata et al. (2018) [91]	USA (Pennsylvania)	Quasi-experimental, pretest–posttest without control	Training type: mandatory Setting: primary care (ambulatory clinic) Clinical staff: resident physician and faculty preceptor (*N* = 129)	Population focus: all LGBTQ + populations Topics: LGBTQ + terminology and culture, distinct health needs and care considerations for LGBTQ + individuals, health disparities for LGBTQ + persons, LGBTQ + inclusive clinical practice knowledge and skills, structural and systemic oppression of LGBTQ + people		National LGBTQIA + Health Education Center	Approach: Multimodal Training mode: in-person (Didactic lecture, group discussion)	3 h	Clinician educator
Walia et al. (2019) [92]	USA (Ohio)	Quasi-experimental, pretest–posttest without control (Data collected with recurrent interventions over 0.5 years)	Training type: mandatory Setting: acute care (perioperative) Clinical staff: physician, nurse, nurse anesthetist, patient care assistant, surgical technician (*N* = 169)	Population focus: LGBTQ + youth Topics: LGBTQ + terminology and culture, distinct health needs and care considerations for LGBTQ + individuals, health disparities for LGBTQ + persons			Approach: Single modality Training mode: in-person (Didactic lecture)		Director of the LGBTQ + health initiative
Weeks et al. (2018) [93]	USA (California)	Quasi-experimental, pretest–posttest without control (Data collected with recurrent interventions over 2 years)	Training type: voluntary Setting: community (foster family agency, group home, adoption agency) Clinical staff: clinician, social worker Non-clinical staff: group home staff (*N* = 455)	Population focus: all LGBTQ + populations Topics: LGBTQ + terminology and culture, structural and systemic oppression of LGBTQ + people, strategies to create a welcoming and inclusive environment for LGBTQ + clients, bias assessment and mitigation	Implementation framework		Approach: Multimodal Training mode: in-person (Didactic lecture, coaching meeting)	3 h	Trainer (not specified)
White-Hughto et al. (2017) [94]	USA (Connecticut and Massachusetts)	Quasi-experimental, pretest–posttest without control (3-month follow-up)	Training type: voluntary Setting: community (correctional) Clinical staff: counselor, nurse, nurse practitioner, physician, psychiatrist, social worker, case manager Non-clinical staff: administrator, optometrist (*N* = 34)	Population focus: transgender individuals who are incarcerated Topics: LGBTQ + terminology and culture, distinct health needs and care considerations for LGBTQ + individuals, health disparities for LGBTQ + persons	The theory of planned behavior and the information, motivation, and behavioral skills		Approach: Multimodal Training mode: in-person (Didactic lecture, group discussion, role-play simulation, case study)	1.5 h	Non-LGBTQ + trainers
Wyckoff (2019) [95]	USA (Alabama)	Quasi-experimental, pretest–posttest without control	Training type: voluntary Setting: acute care Clinical staff: nurse (*N* = 30)	Population focus: all LGBTQ + populations Topics: LGBTQ + terminology and culture, health disparities for LGBTQ + persons, LGBTQ + inclusive clinical practice knowledge and skills	Culture care theory of diversity and universality	National LGBTQIA + Health Education Center	Approach: Single modality Training mode: online (Didactic lecture using online module)	0.5 h	

^*^ Abbreviations: *USA* The United States of America, *LGBTQIA* lesbian, gay, bisexual, transgender, queer, intersex, asexual, *HIV* human immunodeficiency virus

Thirty-three studies (75%) were published between 2017 and 2023. The earliest study was from 2002. Studies were clustered into four types of design: randomized controlled trial (*n* = 1), quasi-experimental pretest–posttest without control (*n* = 39), posttest only with control (*n* = 1) and posttest only without control (*n* = 3).

### Methodological quality

Scores from the JBI Checklists [50, 51] of the 44 studies were moderate overall, ranging from 55.6% to 77.8%, with an average of 75.7% and a standard deviation of 5.3%. 39 studies had low risk of bias (≥ 70%); five studies had moderate risk of bias (50–69%). The primary reasons for risk of bias for quasi-experimental studies were (1) the absence of control groups, and (2) the outcomes being measured at single time points pre- and post-intervention, which limits exploration of mechanisms other than the proposed *cause* (the intervention) driving the observed *effect* [50]. The main reason for the risk of bias in the randomized controlled trial study was the lack of blinding.

### Sample characteristics and settings

Sample sizes ranged from 6 to 2,850. 27.3% of the studies were conducted on relatively small samples (n ≤ 30); 63.6% on moderate sized samples (31 ≤ n ≤ 300); and 9.1% on large samples (≥ 301). Health professionals were categorized into five work settings: primary care clinics (*n* = 9), acute care hospitals (*n* = 9), long-term care facilities (*n* = 2), community facilities (*n* = 3), and mixed healthcare settings (*n* = 21), which included participants recruited from more than one category. Three community facilities included a senior living center [66], a group home/foster family agency [93] and a correctional facility [94]. Three studies [70, 77, 88] were conducted within the military health system.

Training programs were conducted in three different settings: voluntary (*n* = 33), mandatory (*n* = 10) such as a part of residency programs, and mixed voluntary and mandatory (*n* = 1) when researchers recruited multiple sites where some facilities mandated the training, while others did not. Among 44 studies reviewed, 30 studies included personnel from multiple disciplines. 14 engaged health professionals from a single discipline, including physicians (*n* = 7), nurses (*n* = 4), clinical psychologists (*n* = 1), pharmacist (*n* = 1) and social workers (*n* = 1). 22 studies included only clinical staff, and 22 engaged both clinical and non-clinical employees, such as front desk workers, administrators, and executives.

### Theoretical framework

The majority of studies did not explicitly mention a theoretical framework. In 21 studies, various theoretical frameworks were used to justify or provide a rationale for the study, to design the training, to select outcomes, and/or to interpret the results. Minority stress theory [104] was most frequently utilized (*n* = 5). Three studies [59, 69, 96] used the concept of cultural humility in conjunction with cultural competence.

Theoretical frameworks were used to address (1) cultural and interpersonal components: the model of cultural competence in healthcare delivery [106], cultural care theory of diversity and universality [110], intergroup contact theory [111], the theory of interpersonal relations [115]; (2) stigma components: minority stress theory [104], fundamental cause theory [116], intersectionality [108]; (3) behavioral components: the theory of planned behavior [107], information, motivation, and behavioral skills [112, 117], a situated information-motivation-behavioral skills model of care initiation and maintenance [113], motivational interviewing [109], change theory [114]; (4) learning components: adult learning theory [105], transformative learning theory [118]; and (5) intervention design components: the framework of interprofessional collaborative practice [119], implementation framework [120], the model of four levels of training evaluation [121].

## Training characteristics

### Training topics

Only 15 studies explicitly mentioned educational resources from LGBTQ + health-related national organizations which they used to develop training contents, including National LGBTQIA + Health Education Center (*n* = 8), National LGBT Cancer Network (*n* = 5), National Resource Center on LGBTQ + Aging (*n* = 1), GLMA: Health Professionals Advancing LGBTQ Equality (*n* = 2).

Whereas 25 studies included topics broadly related to LGBTQ + population, 19 studies covered contents regarding specific subpopulations in the LGBTQ + community: lesbian and gay individuals (*n* = 2), lesbian and bisexual women (*n* = 1), LGBTQ + youth (*n* = 3), LGBTQ + older adults (*n* = 5), LGBTQ + veterans (*n* = 2), transgender and gender-nonconforming persons (*n* = 5), and transgender individuals who are incarcerated (*n* = 1). Training topics were categorized into nine groups, with each study offering one or more of the nine topics: LGBTQ + terminology and culture (*n* = 33), structural and systemic oppression of LGBTQ + people (*n* = 13), intersectionality (*n* = 8), distinct health needs and care considerations for LGBTQ + individuals (*n* = 25), health disparities for LGBTQ + persons (*n* = 36), LGBTQ + inclusive clinical practice knowledge and skills (*n* = 23), bias assessment and mitigation (*n* = 4), strategies to create a welcoming environment for LGBTQ + clients (*n* = 12), and unique lived experiences of LGBTQ + persons (*n* = 13).

Of 13 studies that addressed unique lived experiences of LGBTQ + persons, six studies [62, 63, 65, 74, 75, 86] utilized panel presentations during which LGBTQ + individuals shared their stories; six studies [53, 60, 78, 79, 81, 85] employed videos or documentaries to bring the voices and perspectives of LGBTQ + individuals into the training; and one study [77] utilized both a panel presentation and a documentary video.

### Training modalities

The training programs were delivered through online means (*n* = 7), in-person sessions (*n* = 33), or a combination of both with online lectures and in-person activities (*n* = 4). Training modalities coalesced into four groups: multiple modalities with (*n* = 9) and without simulation (*n* = 25), and single modality with simulation (*n* = 1) and with didactic lectures (*n* = 9). Of the studies utilizing simulation (*n* = 10), each employed one of three strategies: standardized patient (*n* = 1), video-based (*n* = 1), and role-play (*n* = 8). Four studies [69, 82, 85, 94] incorporated anti-bias sessions into their training. Duration of trainings were reported in 37 studies, ranging from 0.5 h to 14.5 h, with an average of 3.2 h.

Three studies [55, 67, 75] collaboratively worked with community organizations and provided community-based interventions, engaging the public together with health professionals. The first [55] used an informative session used to make the public aware of LGBTQ + social issues and health disparities. The second [67] hired a cast of actors and provided a live theatrical format for its education. The third [75] recruited healthcare providers and LGBTQ + people and employed a storytelling modality at a community event where they shared their lived experiences about acceptance, resilience and the power dynamic between healthcare providers and LGBTQ + patients.

### Trainers

Most trainers were content experts who were educators, clinicians, or researchers. Ten studies employed LGBTQ + community members as co-trainers. Three [57, 67, 96] employed an LGBTQ + trainer with experience in training delivery; five [65, 74, 75, 77, 86] facilitated LGBTQ + individuals to share their lived experiences and answer the questions from health professionals; and two [62, 63] utilized LGBTQ + persons as both training experts and panelists.

## Measurement characteristics

### Time interval for measurement

Outcome measurement occurred both before and after educational interventions in 40 studies. Four studies [55, 71, 72, 75] measured outcomes after interventions only without baseline assessments. Most studies (*n* = 39) measured outcomes immediately after training, with the remaining five studies measuring outcomes between six weeks and six months post training. In two studies [94, 96], follow-up measurement, in addition to pre- and post-intervention, was conducted to assess retention.

### Measurement instruments

Fifteen studies utilized multiple instruments to measure outcome variables, and 29 studies used a single tool. The use of measurement instruments was grouped under the following five categories: (1) studies utilizing a single author-developed measurement tool (*n* = 18); (2) studies employing a single existing tool with adaptation (*n* = 4) or (3) without adaptation (*n* = 7); and (4) studies using multiple instruments, including author-developed tools and existing tools (*n* = 8) or (5) only existing tools (*n* = 7). The most frequently used existing instrument was the Gay Affirmative Practice (GAP) scale (*n* = 5) [122]. However, this tool was often adapted to add transgender-relevant items, because it was originally developed to measure practitioners’ behaviors and beliefs when caring only for cisgender lesbian or gay patients. Four studies [59, 69, 73, 90] adapted measurement tools originally developed for health science students.

Of the 44 studies reviewed, 15 reported psychometric properties (e.g., reliability or validity) for all tools used; 29 studies did not report reliability or validity for at least one instrument. Table 6 provides details about measurement instruments in each study and their reported reliability with Cronbach’s alpha scores and/or validity.

**Table 6:** Summary of measurements and outcomes

Author (Year), Country	Measurement Instrument with Reliability and/or Validity	Training Target	Key Finding	Effect Size	Reported Impact of LGBTQ + Cultural Competency Trainings						
					**Cultural Competence Constructs**				**Outcomes unrelated to Cultural Competence Constructs**		
					**Knowledge**	**Skill**	**Attitude**	**Behavior**	**Confidence/ Preparedness**	**Self-Efficacy**	**Comfort Level**
Barrett et al. (2021), USA [53]	Lesbian, Gay, Bisexual, and Transgender Development of Clinical Skills Scale (LGBT-DOCSS) [123] with reported validity	knowledge about LGBTQ + communities	Increased perceived knowledge about LGBTQ + communities (Mean ± SD: 5.3 ± 0.7 → 6.1 ± 1.2, *p* = 0.003)	*g* = 0.83 (Large)	**⇑⇑**		** → **		**⇑⇑**		
		Attitudinal awareness regarding LGBTQ + people	No significant change in attitudinal awareness regarding LGBTQ + people (Mean ± SD: 6.7 ± 0.4 → 6.8 ± 0.4, *p* = 0.33)	*g* = 0.25 (Small)							
		Clinical preparedness to work with LGBTQ + patients	Enhanced clinical preparedness to work with LGBTQ + patients (Mean ± SD: 4.1 ± 1.3 → 5.2 ± 1.1, *p* = 0.001)	*g* = 0.91 (Large)							
	Author-developed survey No report about reliability or validity	Knowledge about LGBTQ + health in dermatology	Increased objective/factual knowledge about LGBTQ + health in dermatology and (Mean ± SD: 16.9 ± 2.7 → 18.5 ± 3.1, *p* = 0.048)	*g* = 0.54 (Medium)							
Bristol et al. (2018), USA [54]	The Ally Identity Measure tool [124] (3 subscales: knowledge and skills, openness and support, and awareness of oppression) with reported reliability with a Cronbach’s alpha of 0.76 to 0.88)	Attitudes toward LGBTQ + persons (*openness* toward LGBTQ + people and *support* toward LGBTQ + individuals)	No significant change in mean scores in the openness and support subscale (Mean ± SD: 24.9 ± 4.3 → 26.6 ± 4.1, *p* = 0.062)	*g* = 0.41 (Small)	^**⇑⇑**^	^**⇑⇑**^	^**→**^				
		Knowledge of the LGBTQ + community and LGBTQ + health	Improved mean scores in the knowledge subscale (Mean ± SD: 23.6 ± 5.3 → 29.2 ± 4.6, *p* < 0.05)	*g* = 1.12 (Large)							
		Skills to work with LGBTQ + patients	Improved mean scores in the skills subscale (Mean ± SD: 23.6 ± 5.3 → 29.2 ± 4.6, *p* < 0.05)	*g* = 1.12 (Large)							
		Awareness of oppression of LGBTQ + communities	Increased mean scores in the oppression awareness subscale (Mean ± SD: 15.5 ± 2.4 → 16.8 ± 2.6, *p* < 0.05)	*g* = 0.52 (Medium)							
Craig et al. (2015), Canada [55]	Adapted from the Lesbian, Gay, Bisexual Knowledge and Attitudes Scale for Heterosexuals (LGB-KASH) [125] with reported reliability with a Cronbach’s alpha of 0.89	Knowledge about LGB youth	Mean scores of participants’ self-assessed knowledge increase (3.59 out of 4) and self-assessed improvement in skills (3.44 out of 4)		^**↑**^	^**↑**^	^**↑**^				
		Skills to work with LGB youth									
		Intention to support LGB youth	After training, participants’ responses for intention to support LGB youth as “Yes” and “No” were 79.5% and 20.5% respectively								
Donaldson et al. (2019), USA [56]	Lesbian, Gay, Bisexual Adapted from the Knowledge and Attitudes Scale for Heterosexuals (LGB-KASH) [125] No report about reliability or validity	Knowledge of the LGBTQ + community and health for LGBTQ + veterans	Increased perceived and objective/factual knowledge about the LGB community (Mean ± SD: 4.36 ± 1.40 → 5.70 ± 0.86, *p* < 0.001)	*g* = 1.17 (Large)	^**⇑⇑**^	^**→**^	^**→**^				
			Increased perceived and objective/factual knowledge about the transgender community (Mean ± SD: 4.32 ± 1.36 → 5.75 ± 1.16, *p* < 0.001)	*g* = 1.11 (Large)							
	Lesbian, Gay, Bisexual Knowledge and Attitudes Scale for Heterosexuals (LGB-KASH) [125] with reported reliability with a Cronbach’s alpha of 0.91 Attitudes Toward Transgender Individuals Scale (ATTIS) [126] with reported reliability with a Cronbach’s alpha of 0.91	Attitudes toward LGBTQ + people	No significant difference between pre- and post-assessments								
	Adapted from Johnson and Federman (2014) [127] with reported reliability with a Cronbach’s alpha of 0.44 to 0.85)	Skills to work with LGBTQ + people	No significant difference between pre- and post-assessments								
Donisi et al. (2020), Belgium, Italy, Poland, UK [57]	Author-developed survey No report about reliability or validity	Knowledge of the LGBTQ + community and LGBTQ + health	Increased objective/factual knowledge about the LGBTQ + community and LGBTQ + health (Median/IQR: 5/4.0–6.0 → 7/5.0–8.0, *p* < 0.001)		^**⇑⇑**^		^**→**^				
		Attitudes toward LGBTQ + people and working with LGBTQ + patients	Improved attitude scores without statistical significance. A “willingness” score was lower than “acknowledgement” and “self-competence” scores								
Felsenstein (2018), USA [58]	Author-developed survey with reported validity	Knowledge of the LGBTQ + community and LGBTQ + health	Increased objective/factual knowledge about the LGBTQ + community and LGBTQ + health (Mean ± SD: 6.3 ± 2.8 → 9.6 ± 2.7, *p* = 0.033)	*g* = 1.14 (Large)	^**⇑⇑**^				^**↑**^		
	Author-developed survey No report about reliability or validity	Perceived preparedness to work with LGBTQ + patients	After training, 72% of staff reported that they were more prepared to provide care to LGBTQ + patients								
Frasca et al. (2019), USA [59]	Adapted from Hayes et al. (2015) [128] No report about reliability or validity	Comfort in HIV prevention topics	Improved comfort level taking a sexual history with LGBTQ + patients (Mean 3.5 → 4.3, *p* < 0.05), initiating a safe sex discussion with LGBTQ + patients (Mean 3.0 → 4.2, *p* < 0.05), and initiating a PrEP discussion (Mean 2.5 → 4.1, *p* < 0.05)						^**↑**^		^**⇑⇑**^
		Confidence in managing sexual health issues with LGBTQ + patients	95% of respondents agreed they felt better prepared to diagnose and manage real-life patients with similar complaints								
Gendron et al. (2013), USA [60]	Author-developed survey No report about reliability or validity	Awareness of aging LGBTQ + people’s healthcare issues	Increased awareness of LGBTQ + persons’ healthcare issues (Mean ± SD: 0.52 ± 0.50 → 0.93 ± 0.26, *p* < 0.001)	*g* = 1.08 (Large)	^**⇑⇑**^						^**⇑⇑**^
		Level of comfort working with an LGBTQ + older adult	Improved comfort level working with LGBTQ + older adults (Mean ± SD: 4.41 ± 0.98 → 4.62 ± 0.77, *p* < 0.001)	*g* = 0.24 (Small)							
Grova et al. (2021), USA [61]	The Ally Identity Measure tool [124] (3 subscales: knowledge and skills, openness and support, and awareness of oppression) with reported reliability with a Cronbach’s alpha of 0.76 to 0.88)	Knowledge of the LGBTQ + community and LGBTQ + health	Training had a significant effect on an improvement in knowledge and skills (*p* = 0.024)	ω^2^ = 0.2 (Large)	^**⇑⇑**^	^**⇑⇑**^	^**⇑⇑**^				
		Skills to work with LGBTQ + patients	Training had a significant effect on an improvement in knowledge and skills (*p* = 0.024)	ω^2^ = 0.2 (Large)							
		Attitudes toward LGBTQ + persons (*openness* toward LGBTQ + people and *support* toward LGBTQ + individuals)	Training had a significant effect on an improvement in openness and support (*p* = 0.042)	ω^2^ = 0.044 (Small)							
		Awareness of oppression of LGBTQ + communities	Training did not have a significant effect on an improvement in awareness of oppression								
Hanssmann et al. (2008), USA [62]	Adapted survey from the Cultural Competency Self-Assessment Questionnaire [129] No report about reliability or validity	Knowledge about clinically and culturally competent care for transgender patients	Showed a 0.6-point increase in perceived knowledge about care for transgender patients (*p* < 0.05)		^**⇑⇑**^						
Hanssmann et al. (2010), USA [63]	Adapted survey from the Cultural Competency Self-Assessment Questionnaire [129] with reported reliability with a Cronbach’s alpha of 0.45 to 0.79) Reported this tool is not validated	Knowledge of transgender and gender non-conforming communities, service delivery/practice, resources, and linkages for the communities	Increased perceived knowledge about the provision of care to transgender and gender non-conforming patients (Mean ± SD: 2.26 ± 0.53 → 1.79 ± 0.53, *p* < 0.05)	*g* = 0.88 (Large)	^**⇑⇑**^			^**⇑⇑**^			
		Behavior consciousness of transgender and gender non-conforming communities	Improved behavior consciousness of working with transgender and gender non-conforming clients (Mean ± SD: 2.42 ± 0.66 → 1.68 ± 0.66, *p* < 0.05)	*g* = 1.11 (Large)							
Hardacker et al. (2014), USA [64]	Author-developed survey No report about reliability or validity	Knowledge of the LGBTQ + elder community and health for LGBTQ + older adults	Increased objective/factual knowledge about the LGBTQ + elder community in the nursing home/home health care group (Mean ± SD: 62.5 ± 18.2 → 67.3 ± 15.6, *p* < 0.01)	*g* = 0.28 (Small)	^**⇑⇑**^						
			Increased objective/factual knowledge about the LGBTQ + elder community in the hospital/educational setting group (Mean ± SD: 82.3 ± 18.6 → 89.8 ± 12.5, *p* < 0.01)	*g* = 0.48 (Small)							
			Increased objective/factual knowledge about health disparities for LGBTQ + older adults in the nursing home/home health care group (Mean ± SD: 55.9 ± 21.1 → 66.8 ± 22.7, *p* < 0.01)	*g* = 0.49 (Small)							
			Increased objective/factual knowledge about health disparities for LGBTQ + older adults in the hospital/educational setting group (Mean ± SD: 73.5 ± 21.1 → 90.4 ± 13.6, *p* < 0.01)	*g* = 0.97 (Large)							
Henry (2017), USA [65]	The Sexual Orientation Counselor Competency Scale (SOCCS) [130] with reported validity	Knowledge of the LGBTQ + community	Increased perceived knowledge about the LGBTQ + community (SD 0.46 → 0.63)		^**↑**^	^**↑**^	^**↑**^				
		Skills to work with LGBTQ + patients	Enhanced self-assessed skills to work with LGBTQ + patients (SD 0.994 → 1.006)								
		Attitudes toward LGBTQ + patients	Improved attitudes toward LGBTQ + patients (SD 0.765 → 0.989)								
Holman et al. (2020), USA [66]	Author-developed survey with reported reliability with pretest Cronbach’s alpha of 0.84 and posttest Cronbach’s alpha of 0.76	Knowledge of the LGBTQ + elder community and unique concerns and needs for LGBTQ + older adults	Increased objective/factual knowledge about the LGBTQ + older adults and their specific needs and concerns (Mean ± SD: 4.32 ± 2.86 → 7.56 ± 1.61, *p* < 0.001)	*g* = 1.34 (Large)	^**⇑⇑**^		^**→**^		**⇓⇓**		
	Adapted survey from (LaMar & Kite, 1998) [131] with reported reliability with pretest Cronbach’s alpha of 0.94 and posttest Cronbach’s alpha of 0.80	Attitudes toward LGBTQ + elders	No significant change in attitudes toward LGBTQ + older adults (Mean ± SD: 4.42 ± 1.56 → 4.27 ± 1.88, *p* = 0.678)	*g* = 0.04 (Trivial)							
	Author-developed survey No report about reliability or validity	Perceived preparedness to work with LGBTQ + older adults	Decreased perceived preparedness to work with LGBTQ + elders (Mean ± SD: 1.87 ± 0.84 → 1.43 ± 0.50, *p* = 0.001)	*g* = 0.61 (Medium)							
Hughes et al. (2016), USA [67]	Author-developed survey No report about reliability or validity	Knowledge or awareness related to the LGBTQ + elder community and unique concerns and needs for LGBTQ + older adults	75% of respondents agreed that their understanding of the unique needs of LGBTQ + older adults increased		^**↑**^				**↑**		
		Preparedness to provide services to LGBTQ + older adults	86% of respondents agreed that they felt better prepared to work with LGBTQ + elders								
Ingraham et al. (2016), USA [68]	Author-developed survey No report about reliability or validity	Knowledge about the barriers to healthcare of overweight and obese LB women	In both academic and clinic format programs, increased knowledge about LB women’s healthcare avoidance based on body size (participants’ response as “agree” 73% → 95%, *p* < 0.05 in academic format training and 77% → 100%, *p* < 0.05 in clinic format training)		^**⇑⇑**^		^**→**^				
		Attitudes toward barriers to healthcare of overweight and obese LB women	In both academic and clinic format programs, did not show statistically significant improvement in attitudes about asking patients’ sexual identity (*p* = 0.18 in academic format training and *p* = 0.2 in clinic format training)								
Jadwin-Cakmak et al. (2020), USA [69]	Adapted from Strong and Folse (2015) [132], Kelley et al. (2008) [133] and Maher and Bower (2015) [134] No report about reliability or validity	Knowledge of the LGBTQ + community and LGBTQ + youth health	Increased objective/factual knowledge about LGBTQ + youth health (Mean ± SD: 7.22 ± 0.71 → 7.82 ± 0.80, *p* = 0.009)	*g* = 0.79 (Medium)	^**⇑⇑**^		^**⇑⇑**^	** → **			
	Adapted from Strong and Folse (2015) [132], Kelley et al. (2008) [133]and Maher and Bower (2015) [134] with reported reliability with pretest Cronbach’s alpha of 0.92 and 6-month follow-up Cronbach’s alpha of 0.91	Attitudes toward LGBTQ + youth	Improved attitudes toward LGBTQ + youth (Mean ± SD: 3.45 ± 0.20 → 3.64 ± 0.17, *p* = 0.003)	*g* = 1.03 (Large)							
	Author-developed survey No report about reliability or validity	LGBTQ + affirming individual practices	No significant change in participants’ reported use of LGBTQ + youth’s preferred names or pronouns (Mean ± SD: 4.72 ± 0.23 → 4.76 ± 0.34, *p* = 0.657)	*g* = 0.14 (Trivial)							
		LGBTQ + affirming clinic-level practices and perceived clinic environment	Improved clinic-level practices (Mean ± SD: 7.22 ± 1.64 → 9.95 ± 1.43, *p* = 0.001)	*g* = 1.78 (Large)							
			No significant changes in clinics’ endorsement of environmental changes for LGBTQ + youth (Mean ± SD: 0.98 ± 0.04 → 1.00 ± 0, *p* = 0.082)	*g* = 0.99 (Large)							
Kaiafas and Kennedy (2021), USA [70]	The Ally Identity Measure tool [124] (3 subscales: knowledge and skills, openness and support, and awareness of oppression) with reported validity and reliability with a Cronbach’s alpha of 0.76 to 0.88	Knowledge of the LGBTQ + community and LGBTQ + health	Improved mean scores in the knowledge and skills subscale (Mean ± SD: 21.78 ± 8.91 → 28.22 ± 7.47, *p* = 0.001)	*g* = 0.79 (Medium)	^**⇑⇑**^	^**⇑⇑**^	^**⇑⇑**^				
		Skills to work with LGBTQ + patients	Improved mean scores in the knowledge and skills subscale (Mean ± SD: 21.78 ± 8.91 → 28.22 ± 7.47, *p* = 0.001)	*g* = 0.79 (Medium)							
		Attitudes toward LGBTQ + persons (*openness* toward LGBTQ + people and *support* toward LGBTQ + individuals)	Increased mean scores in the openness and support subscale (Mean ± SD: 20.17 ± 6.95 → 23.69 ± 7.59, *p* = 0.04)	*g* = 0.48 (Small)							
		Awareness of oppression of LGBTQ + communities	No significant change in mean scores in the oppression awareness subscale (Mean ± SD: 13.28 ± 3.81 → 14.14 ± 4.06, *p* = 0.36)	*g* = 0.22 (Small)							
Kauth et al. (2016), USA [71]	Author-developed survey No report about reliability or validity	Knowledge of the LGBTQ + veteran community and their health	Post training, 94% of respondents (15 out of 16) reported a high level of knowledge (very knowledgeable) about LGBTQ + veteran community and their health		^**↑**^						
Kilicaslan and Petrakis (2023), Australia [72]	Author-developed survey No report about reliability or validity	Knowledge of the LGBTQ + community and their unique needs	After the training, 15.95% of respondents (18 out of 113) strongly agreed that they had adequate knowledge, and 52.21% of respondents (59 out of 113) agreed		^**↑**^						
		Attitudes toward LGBTQ + persons	After the training, 15.95% of respondents (18 out of 113) strongly agreed that they had adequate attitude, and 52.21% of respondents (59 out of 113) agreed								
Lelutiu-Weinberger et al. (2016), USA [73]	Adapted from the Sexual Orientation Provider Competency Scale [130] with reported reliability with a Cronbach’s alpha of 0.90 Adapted from the Clinical Skills and Attitudes Scale [135] originally developed for medical students. No reported about reliability or validity	Knowledge of clinical issues for transgender patients	No significant change in perceived knowledge about clinical issues for transgender patients (Mean ± SD: 26.0 ± 6.2 → 25.4 ± 6.3, *p* > 0.05)	*g* = 0.09 (Trivial)	^**→**^	^**⇑⇑**^	^**⇑⇑**^		^**→**^		
			No significant change in awareness of transphobic practices (Mean ± SD: 9.2 ± 4.0 → 14.0 ± 7.0, *p* < 0.18)	*g* = 0.86 (Large)							
	Adapted from the Sexual Orientation Provider Competency Scale [130] with reported reliability with a Cronbach’s alpha of 0.90	Skills to work with transgender clients	Increased self-assessed skills in working with transgender patients (Mean ± SD: 22.1 ± 6.7 → 28.5 ± 8.4, *p* < 0.01)	*g* = 0.84 (Large)							
	Adapted from the Attitudes toward Transgender Patients Scale [135] Adapted the Modern Homophobia Scale [136] with reported reliability with a Cronbach’s alpha of 0.93	Attitudes toward transgender patients	Decreased negative attitudes toward transgender patients (Mean ± SD: 19.6 ± 7.9 → 17.1 ± 8.4, *p* < 0.05)	*g* = 0.31 (Small)							
			No significant change in transphobia scores (Mean ± SD: 25.0 ± 6.8 → 25.2 ± 7.4, *p* > 0.05)	*g* = 0.03 (Trivial)							
	Adapted from the Contemplation Ladder [137, 138] with reported reliability with a Cronbach’s alpha of 0.30 to 0.76	Readiness to provide care to transgender patients	No significant change in readiness to care for transgender patients without statistical significance (Mean ± SD: 8.6 ± 2.3 → 9.3 ± 1.8, *p* > 0.05)	*g* = 0.34 (Small)							
	Direct observation by the author	Clinic environment change	At the follow-up environmental surveillance, the author identified elements, including LGBTQ + inclusive magazines and brochures, in the waiting area								
Leyva et al. (2014), USA [74]	Author-developed survey with reported validity and reliability with a Cronbach’s alpha of 0.75 Lower scores indicate positive changes	Knowledge of the LGBTQ + elder community and unique concerns and needs for LGBTQ + older adults	Increased perceived knowledge about LGBTQ + older adults and their needs and concerns (Mean ± SD: 20.14 ± 4.01 → 18.19 ± 3.17, *p* < 0.001)	*g* = 0.54 (Medium)	^**⇑⇑**^	^**⇑⇑**^	^**⇑⇑**^				
		Attitudes toward LGBTQ + elders	Improved attitudes toward LGBTQ + older adults (Mean ± SD: 9.13 ± 2.88 → 8.30 ± 2.43, *p* = 0.005)	*g* = 0.21 (Small)							
		Skills to work with LGBTQ + older adults	Increased self-assessed skills to work with LGBTQ + older adults (Mean ± SD: 15.28 ± 3.24 → 12.17 ± 3.00, *p* < 0.001)	*g* = 0.99 (Large)							
Long et al. (2022), USA [75]	Adapted survey from the Jefferson Scale of Physician Empathy [139] and the Jefferson Scale of Patient’s Perceptions of Physician Empathy [140] with no reported reliability or validity	Understanding (empathy) the LGBTQ + people	Post event, 87.5% of respondents agreed that they could better understand their LGBTQ + patients’ emotions, feelings, and concerns		^**↑**^		^**⇑⇑**^	^**⇑⇑**^			
	Gay Affirmative Practice scale [122] with reported validity and reliability with a Cronbach’s alpha of 0.99 (*belief* questions) and 0.96 (*practice* questions)	Belief in appropriate feelings and behaviors when caring for LGBTQ + patients	Participants showed higher belief scores than those who did not attend the event (Mean 71.38 versus 63.90, *p* = 0.024)	*g* = 0.46 (Small)							
		LGBTQ + affirming practices	Participants showed higher practice scores than those who did not attend the event (Mean 70.05 versus 56.12, *p* < 0.001)	*g* = 0.81 (Large)							
McGarry et al. (2002), USA [76]	Author-developed survey (Lower scores indicate positive changes) No report about reliability or validity	Preparedness to care for lesbian and gay patients	Increased preparedness to care for lesbian and gay patients (Mean ± SD: 2.35 ± 0.95 → 1.88 ± 0.70, *p* < 0.001)	*g* = 0.56 (Medium)					^**⇑⇑**^		^**→**^
		Comfort level working with lesbian and gay clients	No significant change in comfort level working with lesbian clients (Mean ± SD: 2.19 ± 0.99 → 1.94 ± 0.74, *p* = 0.06)	*g* = 0.29 (Small)							
			No significant change in comfort level working with gay patients (Mean ± SD: 2.08 ± 0.92 → 1.86 ± 0.53, *p* = 0.07)	*g* = 0.31 (Small)							
Oblea et al. (2022), USA [77]	The Ally Identity Measure tool [124] (3 subscales: knowledge and skills, openness and support, and awareness of oppression) with reported validity and reliability with a Cronbach’s alpha of 0.79 to 0.91)	Attitudes toward LGBTQ + persons (*openness* toward LGBTQ + people and *support* toward LGBTQ + individuals)	Increased mean scores in the openness and support subscale (Mean ± SD: 3.92 ± 0.64 → 4.11 ± 0.64, *p* < 0.001)	*g* = 0.24 (Small)	^**⇑⇑**^	^**⇑⇑**^	^**⇑⇑**^				
		Knowledge of the LGBTQ + community and LGBTQ + health	Improved mean scores in the knowledge subscale (Mean ± SD: 3.14 ± 0.78 → 3.73 ± 0.76, *p* < 0.001)	*g* = 0.67 (Medium)							
		Skills to work with LGBTQ + patients	Improved mean scores in the skills subscale (Mean ± SD: 3.14 ± 0.78 → 3.73 ± 0.76, *p* < 0.001)	*g* = 0.67 (Medium)							
		Awareness of oppression of LGBTQ + communities	Increased mean scores in the oppression awareness subscale (Mean ± SD: 4.175 ± 0.65 → 4.38 ± 0.60, *p* < 0.001)	*g* = 0.26 (Small)							
Pachankis et al. (2022), USA, Canada, Uganda [96]	The Sexual Orientation Counselor Competency Scale (SOCCS) [130] with reported reliability with Cronbach’s alpha of 0.83 to 0.91	Clinical skills in LGBTQ-affirmative cognitive behavioral therapy	The intervention group showed relative improvements in self-reported LGBTQ + cultural competence compared to the control group (*p* < 0.001) Intervention group (Mean ± SE: 3.31 ± 0.17 → 5.05 ± 0.15) Control group (Mean ± SE: 3.30 ± 0.19 → 3.14 ± 0.18)	*g* = 1.23 (Large)	^**⇑⇑**^	^**⇑⇑**^					
	The Multidimensional Cultural Humility Scale (MCHS) [141] with reported reliability with Cronbach’s alpha of 0.68 to 0.75	Cultural humility when working with LGBTQ + clients	The intervention group did not show relative improvements in LGBTQ + cultural humility (*p* > 0.05) Intervention group (Mean ± SE: 76.48 ± 0.90 → 77.34 ± 0.88) Control group (Mean ± SE: 75.28 ± 0.83 → 75.76 ± 1.03)	*g* = 0.48 (Small)							
	Author-developed survey No report about reliability or validity	Knowledge of minority stress theory	The intervention group showed relative improvements in minority stress knowledge compared to the control group (*p* < 0.001) Intervention group (Mean ± SE: 7.02 ± 0.19 → 8.05 ± 0.21) Control group (Mean ± SE: 7.15 ± 0.19 → 7.04 ± 0.26)	*g* = 1.14 (Large)							
	Author-developed survey No report about reliability or validity	Knowledge of general cognitive behavioral therapy and LGBTQ-affirmative cognitive behavioral therapy skills	The intervention group showed relative improvements in LGBTQ-affirmative cognitive behavioral therapy knowledge compared to the control group (*p* < 0.001) Intervention group (Mean ± SE: 5.36 ± 0.24 → 6.77 ± 0.30) Control group (Mean ± SE: 5.48 ± 0.21 → 5.46 ± 0.26)	*g* = 1.38 (Large)							
	Author-developed survey with reported reliability of Cronbach’s alpha of 0.77 to 0.85	Self-reported familiarity with the LGBTQ-affirmative cognitive behavioral therapy skills	The intervention group showed relative improvements in LGBTQ-affirmative skills familiarity compared to the control group (*p* < 0.001) Intervention group (Mean ± SE: 18.30 ± 0.76 → 24.34 ± 0.66) Control group (Mean ± SE: 19.00 ± 0.66 → 19.70 ± 0.76)	*g* = 5.03 (Large)							
		LGBTQ-affirmative kills use	The intervention group showed relative improvements in LGBTQ-affirmative skills use compared to the control group (*p* < 0.001) Intervention group (Mean ± SE: 16.77 ± 0.67 → 22.93 ± 0.72) Control group (Mean ± SE: 17.53 ± 0.66 → 18.46 ± 0.77)	*g* = 4.99 (Large)							
	Author-developed simulated practice assessment	LGBTQ-affirmative cognitive behavioral therapy skills	The intervention group showed relative improvements in LGBTQ-affirmative cognitive behavioral therapy skills compared to the control group (*p* < 0.001) Intervention group (Mean ± SE: 0.11 ± 0.0.2 → 0.24 ± 0.03) Control group (Mean ± SE: 0.12 ± 0.02 → 0.14 ± 0.02)	*g* = 0.12 (Trivial)							
Pelts and Galambos (2017), USA [78]	The Components of Attitudes Toward Homosexuality Scale [131] with reported reliability with a Cronbach’s alpha of 0.79 to 0.91	Attitude toward lesbian and gay older adults	Improved attitudes toward caring for lesbian and gay clients (Mean ± SD: 85.45 ± 12.04 → 87.66 ± 11.54, *p* < 0.001)	*g* = 0.19 (Trivial)			^**⇑⇑**^				
Pepping et al. (2018), Australia [79]	The Modern Homonegativity Scale [142] with reported reliability with a Cronbach’s alpha of 0.85	Reduction of homonegativity	Decreased homonegativity (Mean ± SD: 17.83 ± 5.64 → 16.37 ± 4.60, *p* < 0.004)	*g* = 0.29 (Small)	^**⇑⇑**^	^**⇑⇑**^	^**⇑⇑**^				
	Adapted the Modern Homonegativity Scale with reported reliability with a Cronbach’s alpha of 0.88	Reduction of trans-negativity	Decreased trans-negativity (Mean ± SD: 17.28 ± 5.82 → 16.05 ± 4.74, *p* < 0.001)	*g* = 0.23 (Small)							
	The Lesbian, Gay, and Bisexual Affirmative Counseling Self-Efficacy Inventory (LGB-CSI) [143] with reported reliability with a Cronbach’s alpha of 0.96 (knowledge), 0.95 (advocacy), 0.92 awareness, 0.89 (relationship), and 0.81 (relationship)	Knowledge of the LGBTQ + community and LGBTQ + mental health	Increased perceived knowledge about the LGB people and their mental health (Mean ± SD: 32.51 ± 12.52 → 45.99 ± 13.46, *p* < 0.004)	*g* = 1.04 (Large)							
			Increased perceived knowledge about the transgender people and their mental health (Mean ± SD: 29.27 ± 12.73 → 43.74 ± 14.44, *p* < 0.004)	*g* = 1.07 (Large)							
		Advocacy skills for LGBTQ + patients	Improved self-assessed advocacy skills to work with LGB patients (Mean ± SD: 16.12 ± 7.60 → 21.76 ± 7.85, *p* < 0.004)	*g* = 0.73 (Medium)							
			Improved self-assessed advocacy skills to work with transgender patients (Mean ± SD: 14.23 ± 7.42 → 19.74 ± 8.49, *p* < 0.004)	*g* = 0.69 (Medium)							
		Awareness of health professionals’ own feelings about sexual orientation/gender identity and LGBTQ + patients	Increased awareness of health professionals’ own feelings about sexual orientation/gender identity and LGB patients (Mean ± SD: 19.28 ± 5.47 → 23.00 ± 4.41, *p* < 0.004)	*g* = 0.75 (Medium)							
			Increased awareness of health professionals’ own feelings about sexual orientation/gender identity and transgender patients (Mean ± SD: 18.57 ± 5.70 → 22.92 ± 4.77, *p* < 0.004)	*g* = 0.83 (Large)							
		Assessment of mental health for LGBTQ + people	Enhanced mental health assessment for LGB individuals (Mean ± SD: 12.55 ± 4.44 → 15.70 ± 4.44, *p* < 0.004)	*g* = 0.71 (Medium)							
			Enhanced mental health assessment for transgender individuals (Mean ± SD: 11.78 ± 4.57 → 15.46 ± 4.57, *p* < 0.004)	*g* = 0.81 (Large)							
		Capacity to form a therapeutic relationship with LGBTQ + patients	Improved a capacity to form a therapeutic relationship with LGB patients (Mean ± SD: 10.73 ± 3.50 → 12.64 ± 3.42, *p* < 0.004)	*g* = 0.55 (Medium)							
			Improved a capacity to form a therapeutic relationship with transgender patients (Mean ± SD: 9.51 ± 3.62 → 12.05 ± 3.44, *p* < 0.004)	*g* = 0.71 (Medium)							
Pratt-Chapman (2020), USA [80]	Cultural Competency Assessment (CCA) [144] with reported validity and reliability with a Cronbach’s alpha of 0.92	Cultural awareness and sensitivity for LGBTQ + individuals	No significant change in cultural awareness and sensitivity for LGBTQ + patients (Mean ± SD: 28 ± 3.73 → 28.647 ± 4.80, *p* = 0.430)	*g* = 0.15 (Trivial)	^**→**^		^**⇑⇑**^	^**→**^	^**→**^		
		Cultural behavior toward LGBTQ + persons	No significant change in cultural behavior toward LGBTQ + patients (Mean ± SD: 43.647 ± 10.48 → 48.294 ± 13.33, *p* = 0.055)	*g* = 0.38 (Small)							
	Lesbian, Gay, Bisexual, and Transgender Development of Clinical Skills Scale (LGBT-DOCSS) [123] with reported validity and reliability with a Cronbach’s alpha of 0.86	Attitude toward caring for LGBTQ + patients	Improved attitudes toward caring for LGBTQ + clients (Mean ± SD: 23.2 ± 4.67 → 24.8 ± 4.11, *p* = 0.046)	*g* = 0.35 (Small)							
		Clinical preparedness to work with LGBTQ + people	No significant change in clinical preparedness to work with LGBTQ + individuals (Mean ± SD: 13.5 ± 6.96 → 15.5 ± 6.71, *p* = 0.117)	*g* = 0.28 (Small)							
		Knowledge of the LGBTQ + community and LGBTQ + health	No significant change in perceived knowledge of the LGBTQ + community and LGBTQ + health (Mean ± SD: 12.063 ± 3.87 → 13.313 ± 2.75, *p* = 0.116)	*g* = 0.37 (Small)							
Pratt-Chapman (2021), USA [81]	Gay Affirmative Practice scale [122] with no report about reliability or validity	Gay-affirming behaviors (baseline assessment only)	The lowest mean score from self-reported baseline gay-affirming behaviors was “I help clients identify their internalized homophobia”		^**⇑⇑**^		^**→**^		^**⇑⇑**^		
	Lesbian, Gay, Bisexual, and Transgender Development of Clinical Skills Scale (LGBT-DOCSS) [123] with no report about reliability or validity Lower scores indicate greater competence	Clinical preparedness to work with LGBTQ + people	Improved clinical preparedness to work with LGBTQ + persons (Mean ± SD: 20.76 ± 5.514 → 15.51 ± 4.377, *p* < 0.001)	*g* = 1.04 (Large)							
		Knowledge of the LGBTQ + community and LGBTQ + health	Expanded perceived knowledge of the LGBTQ + community and LGBTQ + health (Mean ± SD: 7.58 ± 2.996 → 5.59 ± 1.930, *p* < 0.001)	*g* = 0.79 (Medium)							
		Attitude in caring for LGBTQ + patients	No significant change in attitudes toward caring for LGBTQ + patients (Mean ± SD: 8.43 ± 2.084 → 8.80 ± 3.7, *p* = 0.544)	*g* = 0.13 (Trivial)							
Pratt-Chapman et al. (2022), USA [82]	Author-developed survey (QUIRKS: Queering Individual and Relational Skills and Knowledge Scales) Lower scores indicate more LGBTQ + affirming care No report about reliability or validity	Knowledge of affirming care for LGBTQ + patients	Expanded perceived knowledge of affirming care for LGBTQ + patients (Mean ± SD: 6.86 ± 3.44 → 5.86 ± 3.03, *p* = 0.043)	*g* = 0.31 (Small)	^**⇑⇑**^		^**→**^	^**⇑⇑**^	^**⇑⇑**^		
		Clinical preparedness to provide affirming care to LGBTQ + people	Improved clinical preparedness to provide affirming care to LGBTQ + persons (Mean ± SD: 9.25 ± 3.99 → 3.13 ± 2.42, *p* = 0.006)	*g* = 1.88 (Large)							
		Clinical behaviors regarding welcoming LGBTQ + individuals	Improved clinical behaviors regarding welcoming LGBTQ + individuals (Mean ± SD: 4.23 ± 3.02 → 2.68 ± 3.14, *p* = 0.022)	*g* = 0.51 (Medium)							
		Attitude toward LGBTQ + care	No significant change in attitudes toward LGBTQ + care (Mean ± SD: 2.64 ± 2.09 → 2.27 ± 2.57, *p* = 0.502)	*g* = 0.16 (Trivial)							
		Environmental cues in clinic	Improved environmental cues in clinic (Mean ± SD: 2.55 ± 2.02 → 1.32 ± 1.84, *p* = 0.018)	*g* = 0.63 (Medium)							
Rhoten et al. (2021), USA [83]	Author-developed survey No report about reliability or validity	Knowledge about the LGBTQ + community	Increased objective/factual knowledge about the LGBTQ + community (*p* < 0.001)		^**⇑⇑**^		^**⇑⇑**^	^**⇑⇑**^		^**⇑⇑**^	
		Attitude toward LGBTQ + people	Improved attitudes toward LGBTQ + persons (*p* < 0.001)								
		Self-efficacy	Enhanced self-efficacy (*p* < 0.001) Only an increase in self-efficacy was significantly correlated with respondents’ improvement in behavioral intention from pretest to posttest (OR = 1.43, 95% CI: 1.22, 1.67, *p* < 0.001)								
		Behavioral intentions	Improved behavioral intentions (*p* < 0.001)								
Rosa-Vega et al. (2020), USA [84]	Author-developed survey with reported validity and reliability with a Kuder-Richardson Formula 20 of 0.653	Knowledge about transgender health and hormone treatments	Increased objective/factual knowledge about the transgender community (Mean: 72.49 → 85.91, *p* < 0.001)	ω^2^ = 0.11 (Medium)	^**⇑⇑**^						
			Increased objective/factual knowledge about transgender health (Mean 50.79 → 66.53, *p* < 0.001)	ω^2^ = 0.26 (Large)							
			Increased objective/factual knowledge about gender-affirming medications (Mean 45.06 → 70.28, *p* < 0.001)	ω^2^ = 0.40 (Large)							
Russell and Corbitt (2022), USA [85]	Author-developed survey No report about reliability or validity	Attitude toward LGBTQ + people	No significant change in attitudes toward LGBTQ + patients (Mean ± SD: 3.65 ± 1.289 → 3.74 ± 1.488, *p* > 0.05)	*g* = 0.06 (Trivial)			^**→**^		^**⇑⇑**^		
		Confidence in providing care to LGBTQ + patients	Increased confidence in providing care to LGBTQ + patients (Mean ± SD: 3.19 ± 1.11 → 4.11 ± 0.828, *p* < 0.001)	*g* = 0.95 (Large)							
Schweiger-Whalen et al. (2019), USA [86]	Gay Affirmative Practice scale [122] with reported validity and reliability with a Cronbach’s alpha of 0.93	Behaviors in practice when caring for LGBTQ + individuals	Improved the GAP (beliefs and behaviors) scores (Mean ± SD: 66.03 ± 6.27 → 70.61 ± 5.94, *p* < 0.001)	*g* = 0.75 (Medium)	^**⇑⇑**^		^**⇑⇑**^	^**⇑⇑**^			
		Beliefs about practice with LGBT + individuals									
	Author-developed survey No report about reliability or validity	Knowledge of the LGBTQ + community and LGBTQ + health	Increased objective/factual knowledge about the LGBTQ + community and LGBTQ + health (Mean ± SD: 6.90 ± 2.18 → 10.18 ± 2.20, *p* < 0.001)	*g* = 1.49 (Large)							
Seay et al. (2020), USA [87]	Author-developed survey No report about reliability or validity	Knowledge of the LGBTQ + community and LGBTQ + health	Increased objective/factual knowledge about the LGBTQ + community and LGBTQ + health (Mean ± SD: 9.6 ± 1.7 → 11 ± 1.0, *p* < 0.001)	*g* = 1.03 (Large)	^**⇑⇑**^		^**⇑⇑**^	^**⇑⇑**^			
	Adapted survey from the Modern Homonegativity Scale (MHS) [142] and the Physicians’ Attitudes Toward Lesbian, Gay, Bisexual, and Transgender Patients Scale (ATLGBTP) [145] with reported validity and reliability with a Cronbach’s alpha of 0.90 and 0.54 respectively	Attitude toward LGBTQ + patients	Improved attitudes toward LGBTQ + patients (Mean ± SD: 22.1 ± 1.8 → 22.8 ± 1.9, *p* = 0.019)	*g* = 0.38 (Small)							
			Decreased homonegativity (Mean ± SD: 23.6 ± 8.6 → 19.8 ± 6.5, *p* = 0.002)	*g* = 0.51 (Medium)							
	Gay Affirmative Practice scale [122] with reported validity and reliability with a Cronbach’s alpha of 0.95	LGBTQ + affirming clinical practices	Increased LGBTQ + affirming clinical practices (Mean ± SD: 51.3 ± 6.7 → 55.8 ± 7.3, *p* < 0.001)	*g* = 0.63 (Medium)							
Shrader et al. (2017), USA [88]	Author-developed survey No report about reliability or validity	Knowledge of LGBTQ + terminology, cultural sensitivity, LGBTQ + health needs and health disparities	Increased objective/factual knowledge of LGBTQ + terminology, cultural sensitivity, LGBTQ + health needs and health disparities		^**↑**^						
Stevenson et al. (2020), USA [89]	Author-developed survey No report about reliability or validity	Knowledge about the endocrine treatment of transgender patients	Did not improve perceived knowledge about the endocrine treatment of transgender patients		^**↓**^	^**↓**^					^**↓**^
		Skills to work with transgender clients	Decreased self-assessed communication skills with transgender patients								
		Comfort level providing care to transgender patients	Decreased comfort level in providing compassionate, appropriate, and effective care to transgender patients								
Traister (2020), USA [90]	Knowledge of Lesbian, Gay, Bisexual, and Transgender People (KLGBT) questionnaire [132] with reported validity and reliability with a Cronbach’s alpha of 0.54	Knowledge of the LGBTQ + community and LGBTQ + health	Increased objective/factual knowledge about the LGBTQ + community and LGBTQ + health (Mean ± SD: 14.18 ± 1.16 → 14.76 ± 0.70, *p* < 0.001)	*g* = 0.62 (Medium)	^**⇑⇑**^		^**→**^				
	Modified Attitudes Toward Lesbians and Gay Men (ATLG) scale [132] & Attitudes Toward Lesbian, Gay, Bisexual and Transgender Patients (ATLGBTP) scale [145] with reported validity and reliability with a Cronbach’s alpha of 0.95 and 0.54 respectively	Attitude toward LGBTQ + patients	No significant change in attitudes toward LGBTQ + persons (Mean ± SD: 3.86 ± 0.58 → 3.94 ± 0.59, *p* = 0.30)	*g* = 0.14 (Trivial)							
Ufomata et al. (2018), USA [91]	Author-developed survey No report about reliability or validity	Knowledge of the LGBTQ + community and LGBTQ + primary care	Increased objective/factual knowledge about primary care for LGBTQ + patients (Mean overall score 42% → 66%, *p* < 0.001)		^**⇑⇑**^				^**⇑⇑**^		
		Perceived confidence in providing primary care to LGBTQ + patients	Increased confidence in implementing gender-neural practices (Mean ± SD: 3.10 ± 0.83 → 3.52 ± 0.57, *p* = 0.0062)	*g* = 0.59 (Medium)							
			No significant change in confidence in eliciting disclosure of gender identity (Mean ± SD: 3.39 ± 0.76 → 3.68 ± 0.60, *p* = 0.0999)	*g* = 0.42 (Small)							
Walia et al. (2019), USA [92]	Author-developed survey No report about reliability or validity	Knowledge of the LGBTQ + community and LGBTQ + adolescent health	Increased objective/factual knowledge about the LGBTQ + community and LGBTQ + adolescent health (Median/IQR: 5/4–6 → 6/4–7, *p* = 0.011)		^**⇑⇑**^						^**→**^
		Level of comfort working with LGBTQ + pediatric patients	No significant improvement in comfort in LGBTQ + culturally competent care (*p* > 0.05)								
Weeks et al. (2018), USA [93]	Author-developed survey with reported reliability and validity	Knowledge of the LGBTQ + community and LGBTQ + adolescent health	Increased objective/factual knowledge about the LGBTQ + community and LGBTQ + adolescent health (Mean ± SD: 15.11 ± 2.76 → 16.74 ± 2.29, *p* < 0.001)	*g* = 0.65 (Medium)	^**⇑⇑**^						
White-Hughto et al. (2017), USA [94]	Author-developed survey No report about reliability or validity	Willingness to provide gender-affirming care	Increased willingness to provide gender-affirming care from pre- to post-test (Mean ± SD: 4.20 ± 0.61 → 4.62 ± 0.49, *p* < 0.001)	*g* = 0.75 (Medium)	^**→**^		^**⇑⇑**^			^**⇑⇑**^	
			Increased willingness to provide gender-affirming care from pre-test to 3-month follow-up (Mean ± SD: 4.20 ± 0.61 → 4.57 ± 0.50, *p* < 0.001)	*g* = 0.66 (Medium)							
	Transgender Knowledge, Attitudes and Beliefs (TKAB) scale [146] with reported validity and reliability with a Cronbach’s alpha of 0.96	Cultural competence (Providers’ knowledge about transgender people and beliefs including transgender stigma and willingness to interact with transgender persons)	Increased cultural competence scores from pre- to post-test (Mean ± SD: 67.54 ± 11.64 → 70.15 ± 10.69, *p* = 0.03)	*g* = 0.23 (Small)							
			Increased cultural competence scores from pre-test to 3-month follow-up (Mean ± SD: 67.54 ± 11.64 → 71.21 ± 10.92, *p* = 0.01)	*g* = 0.33 (Small)							
	Transgender Clinical Competence (TCC) scale [147] with reported validity and reliability with a Cronbach’s alpha of 0.75 to 0.81	Clinical competence (Providers’ general healthcare knowledge and medical gender affirmation knowledge)	No significant change in objective/factual knowledge about general healthcare for transgender patients from pre- to post-test (Mean ± SD: 36.74 ± 4.29 → 37.76 ± 4.36, *p* = 0.08)	*g* = 0.24 (Small)							
			No significant change in objective/factual knowledge about general healthcare for transgender patients from pre-test to 3-month follow-up without statistical significance (Mean ± SD: 36.74 ± 4.28 → 37.71 ± 4.07, *p* = 0.09)	*g* = 0.23 (Small)							
			Increased objective/factual knowledge about medical gender affirmation from pre- to post-test with statistical significance (Mean ± SD: 26.81 ± 3.00 → 29.09 ± 3.32, *p* < 0.001)	*g* = 0.71 (Medium)							
			No significant change in objective/factual knowledge about medical gender affirmation from pre-test to 3-month follow-up without statistical significance (Mean ± SD: 26.81 ± 3.00 → 27.86 ± 2.97, *p* = 0.08)	*g* = 0.35 (Small)							
	Adapted from Thomas and Safer (2015) [148] No report about reliability or validity	Self-efficacy to provide medical gender affirmation therapies (Initiating hormones to transgender men, initiating hormones to transgender women, and continuing hormones for transgender patients)	Increased scores of self-efficacies to initiate hormones for a transgender man from pre- to post-test (Mean ± SD: 2.21 ± 0.74 → 2.53 ± 0.76, *p* = 0.04)	*g* = 0.43 (Small)							
			Increased scores of self-efficacies to initiate hormones for a transgender woman from pre- to post-test with statistical significance (Mean ± SD: 2.38 ± 0.78 → 2.78 ± 0.55, *p* = 0.004)	*g* = 0.61 (Medium)							
			From baseline to 3-month follow-up, no significant change in scores of self-efficacies to initiate hormones for a transgender man (Mean ± SD: 2.21 ± 0.74 → 2.46 ± 0.84, *p* = 0.06)	*g* = 0.32 (Small)							
			From baseline to 3-month follow-up, increased scores of self-efficacies to initiate hormones for a transgender woman (Mean ± SD: 2.38 ± 0.78 → 2.75 ± 0.70, *p* = 0.01)	*g* = 0.49 (Small)							
Wyckoff (2019), USA [95]	Gay Affirmative Practice scale [122] with reported validity and reliability with a Cronbach’s alpha of 0.95	Behaviors in practice when caring for gay and lesbian individuals	Improved the GAP behavior subscale score (Mean ± SD: 47.60 ± 15.75 → 58.07 ± 16.71, *p* < 0.05)	*g* = 0.64 (Medium)			^**→**^	^**⇑⇑**^			
		Beliefs about practice with gay and lesbian individuals	No significant change in the GAP belief subscale score (Mean ± SD: 64.10 ± 8.42 → 66.87 ± 8.15, *p* > 0.05)	*g* = 0.22 (Small)							

^*^ Abbreviations: *SD* standard deviation, *IQR* interquartile range, *PrEP* pre-exposure prophylaxis, *GAP* Gay Affirmative Practice, *LGB* lesbian, gay, bisexual

^*^^⇑⇑ = ^Improvement with statistical significance

^**↑ = **^Improvement (descriptive statistics only)

^**→ = **^No statistically significant change

^**↓ = **^Deterioration (descriptive statistics only)

^**⇓⇓ = **^Deterioration with statistical significance

### Training targets

Most studies measured individual-level changes of participants only, except for two studies [69, 73], which measured both individual-level and organizational-level changes, including changes in clinic environments and clinic-level practices. A variety of terms were used across the studies to describe training targets for health professionals: objective/factual knowledge, perceived knowledge, awareness, preparedness, comfort level, skill, attitude, confidence, affirming practice, openness, support, understanding, empathy, belief, capacity, behavior, self-efficacy, willingness, cultural competence, cultural humility, cultural sensitivity, and clinical competence. After the measurement items and key findings were reviewed, the training targets were segmented into two major categories: cultural competence constructs, including knowledge (*n* = 39), skills (*n* = 12), attitudes (*n* = 27), and behaviors (*n* = 9); and outcomes unrelated to cultural competence, including confidence/preparedness (*n* = 12), self-efficacy (*n* = 2) and comfort level (*n* = 5) An outcomes summary is presented in Table 6.

## Impact of trainings on cultural competence constructs

Based on the four main constructs of cultural competence [32–34], findings from the measurement of knowledge, skills, attitudes, and behaviors, are discussed. Multivariate or stratified analysis was used in eight studies to control potential confounders.

### Knowledge

Among 39 studies measuring change in health professionals’ knowledge, 17 studies measured objective/factual knowledge with multiple choice and/or true/false questions about LGBTQ + populations and their health; 20 studies measured health professionals’ self-perceived knowledge, and two studies [56, 89] measured both objective/factual and perceived knowledge. 28 studies reported statistically significant improvements in knowledge after training; three studies [73, 80, 94] reported no statistically significant changes. In eight studies, outcome data were reported as descriptive, and no inference about the relationship between trainings and knowledge changes was made.

Most studies measured post-training knowledge immediately after training. Four studies measured post-training knowledge several months later. These studies reported disparate results. Three studies [54, 69, 96] reported statistically significant improvements; one study [73] showed no statistically significant changes. In a quasi-experimental study [94] that evaluated knowledge retention by conducting three tests (pretest, posttest, and a three-month follow-up), no significant statistical changes were observed in participants’ factual knowledge regarding LGBTQ + health needs between the pretest and posttest, as well as between the pretest and the three-month follow-up. In a randomized controlled trial study [96] that evaluated knowledge retention three times (pretest, posttest, and a four-month follow-up), the treatment effects on knowledge were sustained at the four-month follow-up. The studies that demonstrated statistically significant improvement in knowledge reported an effect size range of 0.28 to 1.49.

### Skill

Twelve studies assessed change in participants’ skills in working with LGBTQ + patients, including clinical management and LGBTQ + affirming communication skills. Out of the 12 studies, ten studies evaluated participants’ self-assessment of their skill improvements only, while two studies conducted both self-assessment and objective evaluations of participants’ skills [89, 96]. One study [89] utilized a self-report assessment questionnaire and standardized patient-based simulation, while the other study [96] employed a self-report assessment instrument and video-based simulation to measure both self-reported and objective skill evaluations of participants.

Eight studies reported statistically significant improvements in skills after training; one [56] reported no statistically significant skill changes. Three studies reported outcomes with descriptive statistics only. Three studies [54, 61, 96] measured post-training skills several months later instead of immediately after training and both reported statistically significant improvements in participants’ self-assessed skills to work with LGBTQ + patients. In a randomized controlled trial study [96] that evaluated skill retention three times (pretest, posttest, and a four-month follow-up), the treatment effects on skills were sustained at the four-month follow-up. For the studies that showed statistically significant improvement in skill, the reported effect size range was from 0.12 to 1.12.

### Attitude

Changes in heath professionals’ attitudes toward LGBTQ + persons were assessed in 27 studies. 14 studies reported improvements in attitudes with statistical significance. 11 studies reported no statistically significant attitudinal changes. In two studies, outcome data were reported as descriptive, and no inference about the relationship between trainings and attitudinal changes was made.

The only quasi-experimental study with a control group [75] reported that participants showed more positive attitudes toward LGBTQ + affirming practices compared to those who did not participate in the training. The quasi-experimental study which assessed retention of positive attitudes toward transgender persons at three-month follow-up [94] reported statistically significant improvements in participants’ attitudes toward transgender clients compared to baseline assessment. Four studies,which examined post-training attitudinal changes several months later instead of immediately after training reported statistically significant improvements in three studies [61, 69, 75] and no statistically significant changes in one study [54]. The studies that demonstrated statistically significant improvement in attitude reported an effect size range of 0.19 to 1.03.

### Behavior

Nine studies assessed changes in participants’ behaviors toward LGBTQ + affirming practice. Overall, all except for two studies [69, 80] reported statistically significant improvements in participants’ behaviors. The only quasi-experimental study with a control group [75] reported that participants had more positive LGBTQ + affirming behaviors, compared to those who did not participate in the training. The study which measured post-training outcomes six months later instead of immediately after training [69] reported no statistically significant improvement in participants’ LGBTQ + affirming behaviors. The reported effect size range for the studies that showed statistically significant improvement in behavior was from 0.51 to 1.11.

## Impact of trainings on outcomes unrelated to cultural competence constructs

Some studies measured other outcomes unrelated to cultural competence constructs. Three additional outcomes are discussed: confidence/preparedness, self-efficacy, and comfort level.

### Confidence/preparedness

Changes in health professionals’ confidence or preparedness to provide care to LGBTQ + patients were assessed in 12 studies. Six studies reported statistically significant improvements in participants’ confidence/preparedness; two [73, 80] reported no statistically significant changes. Three studies reported outcomes with descriptive statistics only. One study [66] reported statistically significant decreases in nurses’ preparedness to work with LGBTQ + older adults after training. The study which evaluated post-training preparedness to provide care to transgender clients several months later instead of immediately after training [73] reported no statistically significant changes.

### Self-efficacy

Two studies [83, 94] measured change in participants’ self-efficacy, defined as a person’s belief in their capacity to execute behaviors required to yield specific performance attainments [149]. Both studies reported a statistically significant increase in health professionals’ self-efficacy. The study which examined self-efficacy retention at three-month follow-up [94] reported that participants’ self-efficacy to initiate and continue hormones for transgender patients remained increased, compared to baseline assessment.

### Comfort level

Five studies examined changes in health professionals’ comfort level providing care to LGBTQ + clients. Two studies [76, 92] reported statistically significant improvements in participants’ comfort level; two studies reported no statistically significant changes in comfort level. In one study, outcome data were reported as descriptive, and no inference about the relationship between trainings and changes in comfort level was made.

## Discussion

This systematic review assessed studies that quantitatively evaluated the effectiveness of LGBTQ + cultural competency trainings for health professionals. Based on our review, there has been an increased emphasis on LGBTQ + focused cultural competency training programs among health professionals in various healthcare settings within the last five years. Even though direct comparison between studies and estimation of the pooled effect size under meta-analysis were not feasible due to the heterogeneity of training programs, study designs and measured outcomes, the findings of this review highlight the feasibility of LGBTQ + cultural competency trainings for improving the constructs of cultural competence: (1) knowledge of LGBTQ + culture and health, (2) skills to work with LGBTQ + clients, (3) attitudes toward LGBTQ + individuals and (4) behaviors toward LGBTQ + affirming practices.

Our review found that the effect size ranges varied across the four constructs studied. In the context of training and education, interventions that have an effect size greater than 1.0 are considered to be effective [150]. Notably, among the training programs that demonstrated statistically significant improvements, the largest effect sizes were observed in knowledge, while the smallest effect sizes were observed in attitude. Likewise, while almost three-quarters of the studies reported statistically significant knowledge gain, nearly half of the studies that measured changes in health professionals’ attitudes toward LGBTQ + patients reported no statistically significant attitudinal changes. Additionally, studies measuring multiple outcomes reported much smaller effect sizes for attitudinal outcomes compared to other outcomes such as knowledge or skills.

The findings indicate that LGBTQ + cultural competency training can be designed and provided using an interdisciplinary approach and with multiple modalities. These strategies enable health professionals to explore the intricacies of LGBTQ + health and well-being and to identify barriers to providing optimal and individualized care to LGBTQ + clients. Also, the use of multiple pedagogical approaches, including interactive workshops, appears more successful than trainings that use a single strategy to accommodate trainees’ different learning styles and leading to learners’ behavior change [151–154]. The findings of this review additionally highlight the benefit of including LGBTQ + persons as co-trainers to express the diversity of LGBTQ + lived experiences and bring community voices to the trainings. Among studies measuring changes in health professionals’ attitudes toward LGBTQ + individuals, programs that included LGBTQ + co-trainers tended to have statistically significant improvements in trainee attitudes compared to programs which did not include them.

Although cultural competence has been frequently used as a training framework, some scholars and patient advocates nonetheless consider cultural humility to be a more appropriate value than cultural competence for health professionals to develop and carry, as it stresses the significance of providers being open to and curious about individual clients’ experiences, values, and viewpoints, as well as the jeopardy of making assumptions or generalizations based on limited experience or training [30, 155]. However, cultural competence is still valued and serves as a popular training framework in many academic and professional settings because it emphasizes the need for a certain level of education and skill [30, 83], and the term is frequently used as a matter of policy, and in legislative mandates [31]. Recent studies [155, 156] assert that cultural competence and cultural humility are not mutually exclusive, and each serves a pivotal role in practice. Therefore, training programs that incorporate both concepts are needed, and they should be explicit about the values they are prioritizing and designing.

Despite increased LGBTQ + cultural competency training programs for health professionals, there are many underexplored considerations which could strengthen these initiatives. The absence of theoretical framing in most studies is a concern, given that less than half of the reviewed studies exclusively mentioned a theory informing and guiding their work. Even most theory-based studies in our review used theories minimally; very few studies rigorously applied a theory in their rationale, intervention development, selection of outcomes, and interpretation of findings. Theories can provide a foundation for the investigation of relationships, explanation of behavior and prediction of the effect of interventions [157, 158], and theory-based approaches for intervention studies are likely to be more effective than those that are purely empirical or pragmatic [159]. Therefore, a more comprehensive use of theory in research should be considered to increase the quality and effectiveness of interventions. Specifically, trainings that target knowledge, attitudes and behavior can benefit from robust theoretical framing.

It is critical to study the impact of these training programs on patient health outcomes beyond measuring knowledge gain alone. Consistent with other reviews regarding LGBTQ + focused training for health professionals [44, 160], many training programs in our review focused solely on imparting accurate factual information with didactic lectures and only measured knowledge changes. This approach is likely based upon the assumption that once health professionals are well informed of LGBTQ + health issues, they will engage in LGBTQ + affirming behaviors which may result in improved cross-cultural communication and interpersonal relationships with LGBTQ + clients. However, knowledge gain by itself is not predictive of behavior and is insufficient for behavior change [161]. Therefore, training programs need alternative approaches that target more than knowledge gain.

Changes in health professionals’ attitudes and actual behaviors should be prioritized. Attitude is a vital construct contributing to behavior change based on the theory of planned behavior [107]; thus, trainings to improve health professionals’ attitudes toward LGBTQ + persons are essential. The ultimate goals of these trainings should be actual changes in health professionals’ behaviors and skills, which may improve patient-provider interactions and contribute to better patient outcomes and satisfaction for LGBTQ + patients. Despite this, nearly half of the studies that examined the attitudes of health professionals toward LGBTQ + patients did not report statistically significant changes in attitudes. Moreover, all the mandatory training programs that evaluated changes in attitudes did not report statistically significant improvements. Although a 2019 systematic review on training to reduce LGBTQ + related bias for students in health professional curricula [26] had more positive changes in attitude or implicit bias, they were mostly measured anecdotally. Among the studies in our review that incorporated anti-bias sessions into their training [69, 82, 85, 94], only half of them showed statistically significant improvements in attitudes. It is unclear whether inadequate power due to small sample sizes hindered the detection of statistically significant results. However, identifying effective strategies to improve attitudes toward LGBTQ + patients should be prioritized.

The findings may also indicate that a brief exposure to training may not be sufficient to improve one’s attitudes toward LGBTQ + clients, given that training duration was less than 3 h for most studies reporting no improvement in health professionals’ attitudes. This indicates that health professionals could benefit from longer and follow-up LGBTQ + focused trainings, as greater exposure to LGBTQ + patients has been associated with more positive attitudes in previous studies [135, 162]. Although LGBTQ + healthcare equality leaders designated by Healthcare Equality Index [43] tended to be academic medical centers or located in West and Northeast U.S. regions, our findings indicate that health professionals’ unchanged attitudes were not associated with the regions where they practice or the settings in which they work. Therefore, these efforts should be universal. Further, majority of the studies focused on trainee results. Future work on health professionals’ attitudinal and behavioral changes should be correlated to patient-reported experiences, which would more fully evaluate the impact of training programs.

An LGBTQ + focused needs assessment and establishment of clear goals and objectives with the specific audiences should be conducted prior to training. Many studies included both clinical and non-clinical staff, such as administrators, in their training. It is essential to provide high-quality cultural competency training to non-clinical employees, given that they account for more than 30% of healthcare jobs according to U.S. Bureau of Labor Statistics [163] and that they are often the first people with whom LGBTQ + clients interact, establishing the tone for subsequent healthcare encounters. However, delivering content regarding the specific clinical considerations of LGBTQ + patients (e.g., treatment guidelines) to non-clinical staff may not be ideal because it can confuse non-clinical employees about the purpose of training. This may explain why all studies reporting no statistically significant knowledge changes included both clinical and non-clinical staff. Thus, each LGBTQ + cultural competency training program should be designed for its specific audience, with careful assessment of needs and explicit objectives [31].

A rigorous evaluation of training program design is needed. Only one of the studies included a randomization process, and only one quasi-experimental study employed a control group. If a randomized control design is not feasible for practical considerations, a quasi-experimental design or implementation science design may be a suitable alternative. Specifically, the stepped wedge cluster randomized controlled trial, which is commonly employed for the evaluation of service delivery or policy interventions provided at the level of the cluster [164], may be more feasible at the institutional level, as all health professionals in the study design will receive interventions sequentially, with control groups. Also, the reported training duration, the number of participants, and measurement intervals in each training varied across the studies. These differences suggest a need to correlate training duration and size to training outcomes. Our finding that the immediate effects of training outcomes diminished even at a short-term follow-up in most studies suggests that a singular training is insufficient for long-term impacts. Due to a lack of longitudinal assessment, it is also unclear whether positive effects from short-term training programs have long-term viability and sustainability. A single exposure to educational training is unlikely to result in remarkable individual behavior change or institutional change [154, 165–167], follow-up or periodic training sessions with longitudinal evaluation are needed. Robust measurement strategies, including objective evaluation with validated instruments, should also be employed. There is a lack of validated scales to measure health professionals’ cultural competence specifically for LGBTQ + populations. Many studies in our review used author-developed tools without psychometric validation, which is a major threat to the validity of some of the findings. Also, most studies used health professionals’ self-reported evaluations, which may have led to social desirability bias.

The cost-effectiveness of the training programs should be investigated as all training programs requires costs to be developed and provided, and health professionals invests their time in participating in them. However, none of the reviewed studies conducted a cost–benefit analysis of the financial and time costs associated with the trainings. To fill this gap, future research should analyze the financial costs involved in training development and provision, as well as the time costs associated with health professionals’ participation and should compare them to the benefits gained from participation. Moreover, future research should explore the potential long-term benefits of participating in the trainings, such as increased number of LGBTQ + patients’ visits, to understand the overall return on investment. This will provide valuable insights into whether the cost and time spent on the trainings are commensurate with the overall outcomes of participation.

Regardless of how delivery of care is organized, training health personnel can be a crucial first step to raise awareness of LGBTQ + populations and their well-being, and to create a welcoming and inclusive clinical environment. However, it is often the first and only step embarked upon by healthcare entities [4, 168]. Without structural and system-level enhancements regarding diversity, equity and inclusion, cultural competency trainings may not remarkably impact health professionals’ behavioral changes [33, 169]. Beyond staff-wide trainings, efforts toward the incorporation of LGBTQ + cultural competence into all levels of organizational structure, with measurement of institutional changes, are warranted, which could significantly reduce barriers to high quality care for LGBTQ + patients.

## Limitations

This review has several limitations. First, the use of “LGBTQ + ” as an umbrella term risks the homogenization of LGBTQ + populations, thereby potentially obscuring the unique health needs and disparities of LGBTQ + subgroups. Second, by restricting this review to studies that measured training outcomes quantitatively, important insights from foundational qualitative work may have been missed. Third, the findings reported in this review should be considered based upon the quality of the studies. Overall small sample sizes, the lack of psychometric validation of the research instruments and study designs that allow comparisons between groups and longitudinal assessments are threats to the validity of some of the findings. Fourth, this review, including published studies only, may be predisposed to publication bias, which is the tendency for published studies to overrepresent statistically significant findings. Last, as we only included studies published in English and all but three studies were conducted in North American countries, our findings and recommendations may have limited generalizability to other nations with different geographical, historical, cultural, and socio-political contexts.

## Conclusions

Based on our review, there has been a growing number of LGBTQ + specific cultural competency training programs designed for and provided to health professionals in various healthcare settings within the last five years to improve health equity and achieve social justice for LGBTQ + clients. To sustain and advance cultural competency training, it is crucial to establish LGBTQ + inclusive policies and practices within the healthcare system. In addition to developing and providing effective trainings in healthcare settings, it is also necessary to broadly integrate the content and competencies related to LGBTQ + well-being into medical and other allied health science curricula.

Providing LGBTQ + cultural competency trainings may improve patient-provider interactions by enhancing health professionals’ knowledge, skills, attitudes, and behaviors to work with LGBTQ + clients, which may have a positive impact on health outcomes for LGBTQ + individuals. The existing literature indicates that LGBTQ + cultural competency training can include theory-driven, evidence-based, interdisciplinary, and multimodal approaches. Despite the promising results of LGBTQ + cultural competency training in improving health professionals’ cultural competence, there are limitations in study designs, sample sizes, theoretical framing, and the absence of longitudinal assessments and patient-reported outcomes, which call for more rigorous research.

The rising number of state and federal policies that limit LGBTQ + health services emphasizes the pressing need for health professionals to receive culturally responsive training, particularly for interventions that may be required by LGBTQ + individuals, including pregnancy termination or birth control. Policymakers should prioritize funding for research to determine effective training interventions, integrate them into diverse healthcare settings, and guarantee their implementation through continuous evaluations. Moreover, organizations and health systems should prioritize implementing organizational-level changes that foster LGBTQ + inclusive practices to enable access to safe and affirming healthcare services for LGBTQ + individuals.

Nationwide endeavors should be made, concurrent with institutional investments as seen in the reviewed studies, to test effective, evidence-based training programs, with a goal of large-scale integration and standardization of LGBTQ + inclusive care into health systems. Further, a collaborative, international and multi-center study should be conducted to examine how disparate levels of social inclusion and acceptance of LGBTQ + communities in the U.S. and internationally impact LGBTQ + inclusivity in health systems, and to develop transcultural strategies to expand, extend and enhance LGBTQ + inclusive practice worldwide.

## Data Availability

All data generated or analyzed during this study are included in this article.

## Abbreviations

LGBTQ +: Lesbian, Gay, Bisexual, Transgender, Queer or Questioning and others
PRISMA: Preferred Reporting Items for Systematic Reviews and Meta-Analyses
MeSH: Medical Subject Headings
JBI: Joanna Briggs Institute
GAP: Gay Affirmative Practice
